# Increasing Access to Organization Theories for Implementation Science

**DOI:** 10.3389/frhs.2022.891507

**Published:** 2022-06-30

**Authors:** Sarah A. Birken, Linda K. Ko, Mary Wangen, Cheyenne R. Wagi, Miriam Bender, Per Nilsen, Mimi Choy-Brown, Alexandra Peluso, Jennifer Leeman

**Affiliations:** ^1^Department of Implementation Science, Wake Forest School of Medicine, Winston-Salem, NC, United States; ^2^Washington School of Public Health, Health Systems and Population Health, Seattle, WA, United States; ^3^UNC Center for Health Promotion and Disease Prevention, University of North Carolina Chapel Hill, Chapel Hill, NC, United States; ^4^Sue & Bill Gross School of Nursing, University of California, Irvine, Irvine, CA, United States; ^5^Division of Society and Health, Department of Health, Medicine and Caring Sciences, Linköping University, Linköping, Sweden; ^6^School of Social Work, College of Education and Human Development, University of Minnesota, St. Paul, MN, United States; ^7^School of Nursing, University of North Carolina Chapel Hill, Chapel Hill, NC, United States

**Keywords:** organization theory, constructs, propositions, adoption, implementation, sustainment

## Abstract

**Background:**

Organization theories offer numerous existing, highly relevant, yet largely untapped explanations of the organizational dynamics underlying evidence-based intervention (EBI) implementation. Rooted in ideas regarding power, autonomy, and control, organization theories can explain how and why organizations adopt, implement, and sustain EBI use. Although they have gained visibility, organization theories remain underused in implementation research, perhaps due to their inaccessibility to implementation scientists. To improve access to organization theory among implementation scientists, we summarized organization theories with relevance to implementation science.

**Methods:**

Led by the Cancer Prevention and Control Research Network (CPCRN) Organization Theory for Implementation Science workgroup, we employed a modified Delphi process to reach a consensus among 18 experts at the intersection of organization and implementation science regarding organization theories with relevance to implementation science. From texts that described the organization theories, using standardized abstraction forms, two investigators independently abstracted information regarding constructs, propositions regarding how or why constructs might influence implementation, the potential relevance of organization theories' propositions for implementation, and overviews of each theory. The investigators then reconciled discrepancies until reaching consensus. A third investigator reviewed reconciled abstraction forms for accuracy, coherence, and completeness.

**Findings:**

We identified nine organization theories with relevance to implementation science: contingency, complexity, institutional, network, organizational learning, resource dependence, sociotechnical, and transaction cost economics. From the theories, we abstracted 70 constructs and 65 propositions. An example proposition from institutional theory is: “Coercive, mimetic, and normative pressures contribute to organizations…within an organizational field [becoming increasingly similar].” These propositions can be operationalized as levers to facilitate EBI implementation.

**Conclusions:**

To increase use in the field, organization theories must be made more accessible to implementation scientists. The abstraction forms developed in this study are now publicly available on the CPCRN website with the goal of increasing access to organization theories among an interdisciplinary audience of implementation scientists through the CPCRN Scholars program and other venues. Next steps include consolidating organization theory constructs into domains and translating the resulting framework for use among researchers, policymakers and practitioners, aiding them in accounting for a comprehensive set of organization theory constructs thought to influence EBI implementation.

## Introduction

Implementation scientists increasingly acknowledge that evidence-based intervention (EBI) implementation is influenced by organizations' internal and external settings (1, 2). Constructs relating to organizations' internal and external settings (“organization-level constructs”) are reflected in many implementation science theories, models, and frameworks (TMFs). For example, the Consolidated Framework for Implementation Research includes organization-level constructs that could influence implementation such as external policies and incentives (3); Promoting Action on Research Implementation in Health Services includes physical, social, and cultural context that impacts on implementation (4); and the Theory of Innovation Implementation includes access to financial resources as a potential influence on implementation (5–8). Although implementation TMFs include organization-level constructs, they do not comprehensively conceptualize organizations' internal and external settings, which are multifaceted, and implementation TMFs lack nuanced explanations of how and why implementation is influenced by dynamics within and among organizations. Without clear understanding of organization-level constructs' influence on implementation, the potential to leverage organization-level constructs (e.g., restructuring; incentives) to facilitate implementation will remain unrealized (3, 9–11).

Organizations manage implementation by exerting power—i.e., the ability to wield resources to manage implementation in ways that will benefit the organization (e.g., resisting or embracing implementation) (12). To manage implementation, organizations exert many forms of power (e.g., legitimate, coercive, expert), and organizations may exert power horizontally (e.g., strategic relationships within a health system) and vertically (e.g., controlling decisions within government or accrediting bodies). The power that organizations exert to manage implementation can be explained using organization theories. Organization theories describe, explain, and predict complex influences within and across organizations (13). Thus, organization theories have the potential to account for dynamics related to power that organizations exert (e.g., policies, funding, contracts) to manage implementation. Organization theories' history is described in detail elsewhere (14). Briefly, organization theories are rooted in ideas regarding power and associated constructs including structure, autonomy, control. Organization theories explain, for example, how and why organizations come to exist, die, perform as well or as poorly as they do, including organizations' power to adopt, implement, and sustain innovations—or to resist adopting, implementing, or sustaining innovations. Organization theories have been widely used in other fields such as education, public management, and health services research (15–18). Since our initial call for organization theories' use in implementation science (14), the application of organization theory in implementation research has remained limited (19).

Organizational theory remains largely unused and unfamiliar to implementation scientists (14). The field of implementation science has been significantly shaped by the work of many health psychologists, who may view implementation through the lens of psychology, which predominantly focuses on individual-level cognitive constructs thought to influence behavior (e.g., attitudes, beliefs, motivation). These experts have introduced implementation science to a host of psychological theories, which emphasizes the individual's deliberation and rational decision-making process (e.g., Social Cognitive Theory, Theory of Planned Behavior, Health Belief Model). Advances in psychological theories have supported their use in implementation science, for example, through the development of the Theoretical Domains Framework (TDF), which synthesizes 33 psychological theories into 14 domains. Of note, constructs within psychological theories relating to analytical levels beyond individuals (e.g., the TDF's environmental context and resources) remain conceptualized as they relate to individual behavior.

In contrast to the individual focus of psychological perspectives, the analytical level of organization theories is typically the organization or organizational field (e.g., health system). For example, implementation climate, a construct from the Theory of Innovation Implementation, must be measured from the perspective of the organization rather than the individual (20). However, empirical studies in implementation science often collect data from individuals; the challenge of capturing collective-level influences may provide a further explanation as to why organizational theories are not used to the same extent as individual-level theories (21).

To increase the accessibility of organization theories, and thus contribute to more comprehensive, nuanced conceptualization of organization-level constructs in implementation science, our study summarized organization theories and their relevance to implementation science. Our overarching goal is to support the use of organization theories in implementation science by, for example, supporting the selection of strategies that target organization-level constructs that influence implementation.

## Methods

**Figure 1:**
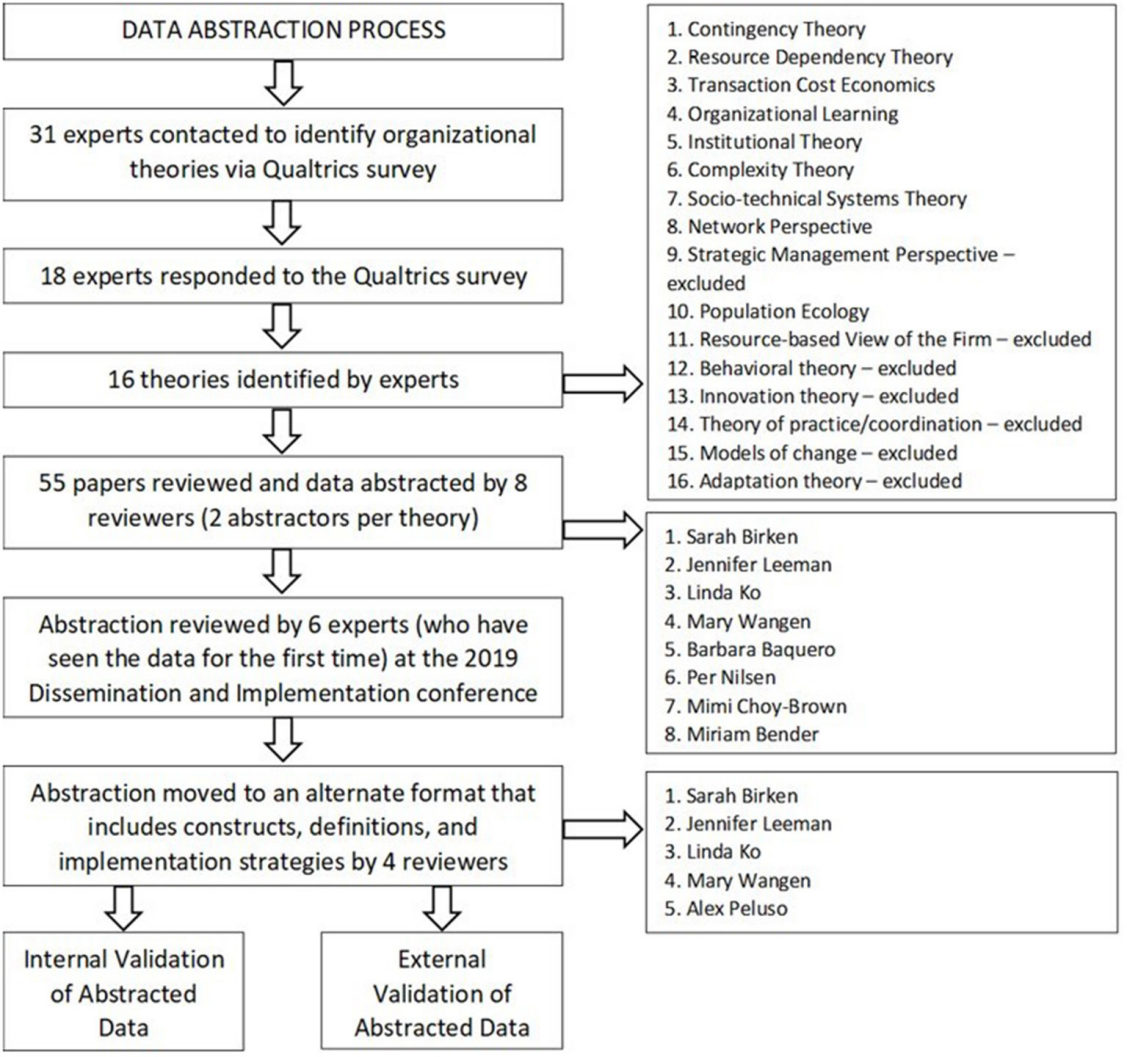
Data abstraction process.

This study was guided by the Cancer Prevention and Control Research Network's (CPCRN) Organization Theory for Implementation Science (OTIS) workgroup (22). CPCRN is a national network of academic, public health, and community partners who work together to reduce the burden of cancer. The OTIS workgroup's overarching mission is to advance the science of implementation by increasing access to organization theory. In meetings that were held monthly or as needed, OTIS workgroup members, who have expertise at the intersection of implementation and organization science, offered perspectives on the purpose, methods, and analysis, and interpretation of the data collected. The study described below employed a modified Delphi process (23–25) to reach a consensus among experts at the intersection of implementation science and organization theory (25) in (1) a survey regarding organization theories relevant to implementation science and foundational texts that described them; (2) a process of developing a standardized form in which to present our findings; and (3) a process of abstracting information from the texts into the form. We describe each of these steps in detail below and in Figure 1.

### Survey to Identify Organization Theories Relevant to Implementation Science

#### Sample and Recruitment

SB and JL, each of whom have expertise at the intersection of implementation and organization science, drew upon their professional networks to identify other experts at the intersection of implementation science and organization theory. SB and JL invited a total of 31 experts working in academic or government institutions in the US, Canada, and Europe to participate in the survey via email, repeating contacts until experts indicated whether they were willing to participate. The survey was deemed not human subjects research by the Institutional Review Board of The University of North Carolina at Chapel Hill.

#### Data Collection

The survey was designed to elicit participants' perspectives on (1) which organization theories may be relevant to implementation science, (2) foundational texts that described organization theories identified as potentially relevant, and (3) texts that demonstrated theories' application in implementation science. To promote understanding of which organization theories may be relevant to implementation science, the survey defined ‘organization theory’ and ‘implementation science’ and listed 12 organization theories that we, as experts at the intersection of organization and implementation science, identified as potentially relevant to implementation science. Participants were asked to review the list and identify theories that should either be added or removed. We also asked participants to select one or more of the theories with which they were most familiar. For each theory they selected, participants were shown a list of texts (61 across the 12 theories) that SB identified as potentially relevant to the theory based on a PhD-level course on organization theory at The University of North Carolina at Chapel Hill. Based on the list of texts, participants were asked to evaluate texts' relevance to describing the theory and to identify additional texts describing the theory generally and it application to implementation science. We administered the survey between July 31, 2018 and November 29, 2018 via Qualtrics (Provo, UT), multifunctional online survey software.

#### Analysis

OTIS workgroup members collaboratively reviewed survey findings. Based on the feedback from survey respondents, theories and texts were added or eliminated.

### Theory Abstraction Form Development

Based on our collective expertise in theory, OTIS workgroup members developed an initial standardized abstraction form. We then hosted a pre-conference meeting at the 12th Annual Conference on the Science of Dissemination and Implementation in Health (26) on December 4th, 2019 to solicit feedback on the form from additional experts in the field. The objective of the meeting was to identify an optimal approach to synthesizing organization theory abstractions into something that would be useful to implementation researchers. Topics of discussion included project background, proposed use, options for distilling the information into a usable format, conceptual level, potential outcomes, and any additional theories not addressed by the survey participants, followed by next steps for the project. Detailed notes were taken during the group discussions and reviewed by the project's leadership team, with the goal of further refining the abstraction form to meet the needs of implementation researchers. We then pilot tested the forms by creating three forms for sample organization theories and applied the theories to three case studies of colorectal cancer screening interventions (27). Institutional Theory, Transaction Cost Economics, and Contingency Theory were applied to the case studies. For each theory, outer setting-level determinants and propositions were defined. The process of applying the forms allowed us to revise them to promote usability. For detailed information on this process, see Leeman et al. (26).

### Abstraction of Data From Theories

The data abstraction process took place over multiple steps (Figure 1). Two OTIS workgroup members abstracted data regarding each theory. First, both members of each pair independently entered data into the abstraction form using information derived from texts identified in the survey as relevant to the theory. Second, pairs met to reconcile discrepancies in abstracted data until consensus was reached. Third, one member of each pair integrated data into a single abstraction form for each theory.

To enhance the validity of the data included in each abstraction form, we recruited experts who participated in the survey to provide feedback on a theory with which they were familiar. We offered participants a $100 honorarium to edit the forms and respond to the following questions: (1) Does the abstracted theory qualify as an organization theory (defined in the survey as, “Organizational theories explain phenomena such as change by explaining relationships between organizations and their environment”)? (2) Does the information on the form reflect the experts' understanding of the theory's tenets? (3) Is any key information missing from the form? (4) Are the claims about the theory's application to implementation science reasonable? We then engaged OTIS workgroup members to provide feedback on the forms with respect to (1) ease of use and quality of formatting, (2) ease of understanding and quality of language use, (3) resonance with members' work, (4) relevance to members' work, (5) relevance to implementation science, and (6) accuracy. The abstractors for each theory were responsible for incorporating feedback into abstraction forms, consulting with the leadership team as needed. Finally, we edited each form for consistency, word choice, and formatting and sought final approval from the OTIS workgroup.

## Results

### Survey

Eighteen experts participated in the survey (58% response rate).

Sixteen theories were identified in the survey: 12 listed in the survey for participants' assessment, and four that participants identified (Figure 1). From the 16 theories, we excluded seven because they represented perspectives rather than theories with falsifiable hypotheses (*n* = 5: Models of Change, Theory of Practice/Coordination, Adaptation Theory, Strategic Management Perspective, and Resource-based View of the Firm) or were not organization theories (*n* = 2: Behavioral Theories and Innovation Theory). The texts that experts identified as relevant to each theory are listed in Table 1.

**Table 1:** Foundational texts by theory – results of the survey.

Complexity theory	Anderson, P, Meyer, A, Eisenhardt, K, Carley, K, Pettigrew, A. Introduction to the special issue: Applications of complexity theory to organization science. *Organization Science*. 1999; 10(3):233−236. https://doi.org/10.1287/orsc.10.3.233
	Byrne, D. Complexity Theory and the Social Sciences: An Introduction. 1998. Rutledge: Oxfordshire, UK.
	Lanham, HJ, Leykym, LK, Taylor, BS, McCannon, CJ, Lindberg, C, Lester, RT. How complexity science can inform scale-up and spread in health care: understanding the role of self-organization in variation across local contexts. *Social Science & Medicine*. 2013; 93:194−202. https://doi.org/10.1016/j.socscimed.2012.05.040
	May, CR, Johnson, M, Finch, T. Implementation, context, and complexity. *Implementation Science*. 2016; 11(141). https://doi.org/10.1186/s13012-016-0506-3
	Miller, WL, Crabtree, BF, McDaniel, R, Stange, KC. Understanding change in primary care practice using complexity theory. *Journal of Family Practice*. 1998; 46(5):369-376. PMID: 9597994
Contingency theory	Drazin, R, & Van de Ven, AH. Alternative forms of fit in contingency theory. *Administrative Science Quarterly*. 1985; 30(4):514-539. https://doi.org/10.2307/2392695
	Galbraith, JR. Organization design: An information processing view. *INFORMS Journal on Applied Analytics*. 1974; 4(3). https://doi.org/10.1287/inte.4.3.28
	Lukas, CV, Holmes, SK, Cohen, AB, Restuccia, J, Cramer, IE, Shwartz, M, Charns, MP. Transformational change in health care systems: An organizational model. *Health Care Management Review*. 2007; 32(4):309-320. doi:10.1097/01.HMR.0000296785.29718.5d
Institutional theory	Donaldson, L. A critique of institutional theory (p79-112). In American anti-management theories of organization: A critique of paradigm proliferation. 1995. Cambridge University Press: Cambridge, UK.
	Fareed, N, Bazzoli, GJ, Mick, SSF, Harless, DW. The influence of institutional pressures on hospital electronic health record presence. *Social Science & Medicine*. 2015; 133:28−35. https://doi.org/10.1016/j.socscimed.2015.03.047
	Greenwood, R, & Hinnings, CR. (1996). Understanding radical organizational change: Bringing together the old and new institutionalism. *The Academy of Management Review*. 1996; 21(4):1022-1054. https://doi.org/10.2307/259163
	Kennedy, MT, & Fiss, PC. Institutionalization, framing, and diffusion: The logic of TQM adoption and implementation decisions among US hospitals. *Academy of Management Journal*. 2009; 52(5):897-918. https://doi.org/10.5465/amj.2009.44633062
	Novotna, G, Dobbins, M, Henderson, J. Institutionalization of evidence-informed practices in healthcare settings. *Implementation Science*. 2012; 7(112). doi:10.1186/1748-5908-7-112
	Oliver, C. Strategic responses to institutional processes. *Academy of Management Review*. 16(1):145-179. https://doi.org/10.2307/258610
	Powell, WW, & DiMaggio, PJ (eds). The New Institutionalism in Organizational Analysis. 1991. Chicago University Press: Chicago, IL.
	Ruef, M, & Scott, WR. A multidimensional model of organizational legitimacy: Hospital survival in changing institutional environments. *Administrative Science Quarterly*. 1998; 43(4):877-904. https://doi.org/10.2307/2393619
	Scott, WR, Ruef, R, Mendel, PJ, Caronna, CA. Institutional Change and Healthcare Organizations: From Professional Dominance to Managed Care. 2000. University of Chicago Press: Chicago, IL.
	Westphal, JD, Gulati, R, Shortell, SM. Customization or conformity? An institutional and network perspective on the content and consequences of TQM adoption. *Administrative Science Quarterly*. 1997; 42(2):366-394. https://doi.org/10.2307/2393924
	Zinn, JS, Weech, RJ, Brannon, D. Resource dependence and institutional elements in nursing home TQM adoption. *Health Services Research*. 1998; 33(2 pt1):261-273. PMCID: PMC1070264
	Zucker, LG. The role of institutionalization in cultural persistence. *American Sociological Review*. 1977; 42(5):726- 43. https://doi.org/10.2307/2094862
Network perspective	Borgatti, SP, & Halgin, DS. On network theory. *Organization Science*. 2011; 22(5). https://doi.org/10.1287/orsc.1100.0641
	Gulati, R & Gargiulo, M. Where do interorganizational networks come from? *American Journal of Sociology*. 1999; 104(5):1439-1493. https://doi.org/10.1086/210179
	Kaluzny, AD, Zuckerman, HS, Rabiner, DJ. Interorganizational factors affecting the delivery of primary care to older Americans. *Health Services Research*. 1998; 33(2 Pt li): 381-401. PMCID: PMC1070357
	Mays, GP, Mamaril, CB, Timsina, LR. Preventable death rates fell where communities expanded population health activities through multisector networks. *Health Affairs*. 2016; 35(11):2005–13. https://doi.org/10.1377/hlthaff.2016.0848
	Meltzer, D, Chung, J, Khalili, P, Marlow, E, Arora, V, Schumock, G, Burt, R. Exploring the use of social network methods in designing healthcare quality improvement teams. *Social Science & Medicine*. 2010; 71(6):1119−1130. https://doi.org/10.1016/j.socscimed.2010.05.012
	Pentland, BT, & Feldman, MS. Narrative networks: Patterns of technology and organization. *Organization Science*. 2007; 18(5):781−795. https://doi.org/10.1287/orsc.1070.0283
	Retrum, JH, Chapman, CL, Varda, DM. Implications of network structure on public health collaboratives. *Health Education & Behavior*. 2013; 40(1 Suppl):13S-23S. https://doi.org/10.1177/1090198113492759
	Ring, PS, & van De Ven, AH. Developmental processes of cooperative interorganizational relationships. *Academy of Management Review*. 1994; 19(1):90–118. https://doi.org/10.2307/258836
	Varda, DM, & Retrum, JH. Collaborative performance as a function of network members' perceptions of success. *Public Performance & Management Review*. 2015; 38(4):632–653. https://doi.org/10.1080/15309576.2015.1031006
	Wholey, DR, Gregg, W, Moscovice, I. Public health systems: A social networks perspective. *Health Services Research*. 2009; 44(5 Pt 2):1842-1862. https://dx.doi.org/10.1111%2Fj.1475-6773.2009.01011.x
	Zuckerman, HS, & D'Aunno, TA. Hospital alliances: Cooperative strategy in a competitive environment. *Health Care Management Review*. 1990; 15(2):21-30. https://www.jstor.org/stable/44950383
Organizational learning	Argote, L. Organizational learning: Creating, retaining and transferring knowledge. (1999) 2013 edition. Springer Science & Business Media: New York, NY
	Cohen, WM, & Levinthal, DA. Absorptive capacity: A new perspective on learning and innovation. *Administrative Science Quarterly*. 1990; 35(1). https://doi.org/10.2307/2393553
	Crossan, MM, Lane, HW, White, RE. An organizational learning framework: From intuition to institution. *Academy of Management Review*. 1999; 24(3):522-537. http://www.jstor.org/stable/259140?origin=JSTOR-pdf
	Harrison, M, & Grantham, S. Learning from implementation setbacks: Identifying and responding to contextual challenges. *Learning Health Systems*. 2018; 2(4):e10068. https://doi.org/10.1002/lrh2.10068
	Huber, GP. Organizational learning: The contributing processes and the literatures. *Organization Science*. 1991; 2(1):88-115. https://psycnet.apa.org/doi/10.1287/orsc.2.1.88
	Lapré, MA, & Nembhard, IM. Inside the organizational learning curve: Understanding the organizational learning process. *Foundations and Trends in Technology, Information and Operations Management*. 2011; 4(1), 1-103. http://dx.doi.org/10.1561/0200000023
	March, JG. Exploration and exploitation in organizational learning. *Organization Science*. 1991; 2(1):71-87. https://doi.org/10.1287/orsc.2.1.71
	Pisano, GP, Bohmer, RMJ, Edmondson, AC. Organizational differences in rates of learning: Evidence from the adoption of minimally invasive cardiac surgery. Management Science. 2001; 47(6):752–768. https://doi.org/10.1287/mnsc.47.6.752.9811
	Singer, SJ, Benzer, J, Hamdan, SU. Improving health care quality and safety: the role of collective learning. *Journal of Healthcare Leadership*. 2015; 7:91-107. doi:10.2147/JHL.S70115
	Tucker, AL, Nembhard, IM, Edmondson, AC. Implementing new practices: An empirical study of organizational learning in hospital intensive care units. *Management Science*. 2007; 53(6). https://doi.org/10.1287/mnsc.1060.0692
Population ecology	Aldrich, HE & Ruef, M. *Organizations Evolving*. 2006 (2^nd^ ed.). SAGE Publications Ltd: London, UK.
Resource-based view of the Firm	Penrose, ET. *The Theory of the Growth of the Firm*. 1995. Oxford University Press: Oxford, England.
Resource dependency theory	Casciaro, T, & Piskorski, M J. Power imbalance, mutual dependence, and constraint absorption: A closer look at resource dependence theory. *Administrative Science Quarterly*. 2005; 50(2):167-199. https://doi.org/10.2189/asqu.2005.50.2.167
	Katz, D, & Kahn, RL. The Social Psychology of Organizations. 1978. Wiley: Ann Arbor, MI
	Pfeffer, J, & Salancik, GR. The external control of organizations: A resource dependence perspective. (1978) 2003 ed. Stanford Business Books: Stanford, CA.
	Zinn, JS, Weech, RJ, Brannon, D. Resource dependence and institutional elements in nursing home TQM adoption. *Health Services Research*. 1998; 33(2 pt1):261-273. PMCID: PMC1070264
Socio-technical systems theory	Holden, RJ, Carayon, P, Gurses, AP, Hoonakker, P, Hundt, AS, Ozok, AA, Rivera-Rodriguez, AJ. SEIPS 2.0: A human factors framework for studying and improving the work of healthcare professionals and patients. *Ergonomics*. 2013; 56(11):1669-1686. doi:10.1080/00140139.2013.838643
	Orlikowski, W J, & Baroudi, JJ. Studying information technology in organizations: Research approaches and assumptions. *Information Systems Research*. 1991; 2(1):1-28. https://doi.org/10.1287/isre.2.1.1
	Riley, B. Systems thinking and dissemination and implementation research. In R. Brownson (Ed.), Dissemination and Implementation Research in Health. 2012 2nd ed. New York: Oxford University Press
	Robertson et al. Implementation and adoption of nationwide electronic health records in secondary care in England: Qualitative analysis of interim results from a prospective national evaluation. *BMJ*. 2010; 341. https://doi.org/10.1136/bmj.c4564 (Published 02 September 2010)
	Walsham, G. Actor-network theory and IS research: Current status and future prospects. In: Lee A.S., Liebenau J., DeGross J.I. (eds) Information Systems and Qualitative Research. 1997. IFIP — The International Federation for Information Processing. Springer, Boston, MA. https://doi.org/10.1007/978-0-387-35309-8_23
Strategic management perspective	Ferlie, E, & Ongaro, E. *Strategic Management in Public Services Organizations: Concepts, Schools and Contemporary Issues*. 2015. Routledge: Oxfordshire, UK.
Transaction cost economics	Bazzoli, GJ, Chan, B, Shortell, SM, D'Aunno, T. The financial performance of hospitals belonging to health networks and systems. *Inquiry*. 2000; 37(3):234-252. https://www.jstor.org/stable/29772899
Behavioral theory	N/A

### Theory Abstraction Forms Development

The initial form draft included the following sections: (1) summary of the theory, (2) key constructs, (3) proposed relationships among key constructs, (3) bounds of theory, (4) implications for implementation, and (5) implications for implementation strategies. After abstracting data from the foundational texts, we revised the forms' sections based on discussion among a total of 12 experts, including OTIS workgroup members and external experts who attended the pre-conference meeting at the 12th Annual Conference on the Science of Dissemination and Implementation in Health. In that process, we identified the need for example applications to implementation science and criticisms, and we renamed the sections for clarity. The resulting final draft of the form included the following sections: (1) theory overview (changed from ‘summary of the theory’), (2) example application to implementation science, (3) constructs and definitions (changed from ‘key constructs’), (4) propositions (changed from ‘proposed relationships among key constructs’), (5) relevance to implementation science (changed from ‘implications for implementation’), and (6) criticisms and/or bounds on the theory (changed from ‘bounds of the theory’).

### Abstraction of Data From Theories

The final abstraction forms describe nine organization theories and their potential relevance to implementation science. Across the theories, we abstracted 70 constructs (Table 2) and 65 propositions. Below, we offer an overview of the data included in each section of the abstraction form and examples of each section from one of the included theories. Supplemental Files - Abstraction Form contains information in each abstraction form organized by section.

**Table 2:** Organization theory constructs and definitions.

**Construct**	**Definition**
**Complexity theory**	
Self-organization	A process whereby local interactions give rise to patterns of organization
Uncertainty	The unpredictability of a system's behavior and its effects
Interdependence	The relationships, connections, and interactions among the parts of a complex system
Feedback loops	A phenomenon characterized by outputs of a system continuously becoming the inputs
Minimum specifications	A few, flexible, simple rules: 1. direction pointing (accounting for past phenomena in future iterations) 2. boundaries (delimitations of the system) 3. resources (means available) 4. permissions [latitude in decision-making; (28)]
Sense making	A social activity through which people assign meaning to experience
**Contingency theory**	
Task	The work that is performed
Task environment	The context where work is performed (both the organizational setting and its wider, socio-political-economic context)
Uncertainty in the task or task environment	The gap between the amount of information that is needed and the amount of information that is available to achieve a given level of performance on a task Factors that may contribute to uncertainty include: ∙ Rate of technical change (how rapidly is the technology required to complete a task changing?) ∙ Lack of information about the availability of resources and stakeholder preferences and demand ∙ Strength/quality of evidence in support of a tasks' impact on intended outcomes
How a task/work is structured: programmed vs. un-programmed coordination (integration)	∙ Programmed coordination: The activities involved in completing a task are specified and codified in advance via (1) rules and programs (i.e., standardization) and (2) centralization of decision making and authority arrangements ∙ Unprogrammed coordination: The activities involved in completing a task are not specified in advance by the organization; activities are worked out by organization members via (1) professionalization deferring to expertise, (2) providing additional time and resources for collaboration, (3) creation of selfcontained tasks, (4) providing real-time data to frontline individuals and teams, and (5) promoting and supporting horizontal coordination and communication
Interdependence	To what degree/extent different actors must interact to complete work.
Differentiation	The extent to which, within an organization, different parts/departments perform different tasks and have different relevant sub-environments.
**Institutional theory**	
Isomorphism	Similar organizational structures and processes (dependent variable)
Coercive pressures	“Formal and informal pressures exerted on organizations by other organizations upon which they are dependent and by cultural expectations in the society within which organizations function“ [(29), p.150]
Mimetic pressures	Influences encouraging organizations to model the behavior of other organizations in their field
Normative pressures	Influences derived from members of an occupation or profession (e.g., physicians) defining the conditions and methods of work
Professionalization	Claims on knowledge among professional groups
**Network perspective**	
Social network	A set of actors (e.g., individuals, organizations) connected by one or more social ties (e.g., advice ties, friendship ties)
Direct ties	Connections in which a single tie spans two actors
Indirect ties	Connections where ties exist between actors but only through other actors
Patterns of relations	Patterns of ties that yield a particular network structure (e.g., structural holes)
Strength	Amount of time, emotional intensity, intimacy (mutual confiding) and reciprocity of the tie
Centrality	The importance of an actor's position in a network structure (e.g., prominence of opinion leaders)
Cohesion	The connectedness or “knitted-ness” of a network
Network density	A measure of cohesion expressed as the number of ties in a network divided by the maximum number of ties that are possible
Constraint	A linkage or other restriction that becomes a limitation and/or an inhibition
Embeddedness	The extent that social ties are forged, renewed, and extended through the community rather than through actors outside the community
Flexibility	The ability of an a network to accommodate change
**Organizational learning**	
Explicit knowledge	Facts and information that can be codified (e.g., in policies and procedures)
Tacit knowledge	Facts, information, and skills that are difficult to codify
Learning process	An interaction of experience (history) and context that produces knowledge
Learning subprocesses	A series of actions associated with the learning process, including: 1. Knowledge creation: knowledge acquired from direct experience of unit (e.g., trial and error experimentation) 2. Knowledge transfer: knowledge transmitted through socialization, education, imitation, professionalization, personnel movement, mergers, acquisitions (Levitt & March) 3. Knowledge retention: knowledge that is embedded in active context (e.g., written policies; job roles) 4. Knowledge search: seeking solutions (in the form of information) for organizational problems
Dominance of organization in field of competitors	The extent to which an organization is perceived to be powerful in relation to its competitors
Complexity of an organization's environment	The extent to which the context in which an organization operates is or is not (1) stable over time and (2) predictable (e.g., customer preferences; availability of resources)
**Population ecology**	
Competition	A process by which “(1) demand for resources exceeds supply; (2) competitors become more similar as standard conditions of competition produce a uniform response; (3) selection eliminates the weakest competitors; and (4) deposed competitors differentiate either territorially or functionally, yielding a more complex division of labor” (30)
Niche/niche width	(The size of) An area in a constraint space in which a population can survive and reproduce itself
Institutional linkages	Relationships created between organization(s) for a cause
Spatial variation	Different values of organizational characteristics across locations
Technology cycles	A sequence of processes that involve technology (i.e., the means, activities, and knowledge to transform materials and inputs into outputs; e.g., human resources)
Selection pressure	External agents that affect an organization's ability to survive in a given environment
Isomorphism	A similarity of processes or structure among organizations
Community interdependence	The extent to which interactions among co-acting sets of organizational/community populations depend on each other
Stability	The extent to which conditions change over time
Population density	The number of organizations in a population (i.e., group of organizations that is distinguishable from other groups)
Internal arrangements	Actions and factors within an organization (e.g., internal politics)
Resource acquisition	The process by which new organization(s) acquire resources
Prior failures	Previous deterioration(s) in an organization's adaptation to its small niche and the associated reduction of resources within the organization
Inertia	Organizational resistance to change
Structure	An organization's goals, authority, strategy, core technology
Specialization	The restricted niche breadth/area of a given organization
Age	The length of an organization's life history
Size	The capacity to carry interactions among resources, constraints and demand
Excess capacity (or slack resources)	Production at a lower scale of output than it has been designed for
**Resource dependency theory**	
Munificence	The availability and accessibility of resources necessary for an organization's development and survival within the external environment
Dynamism	The rate of environmental change or innovation in the external environment
Competition	The number and diversity of stakeholders (competitors, suppliers, and buyers) that an organization needs to consider in formulating strategies (31); perceptions that another organization in the field poses a threat
Power	Dominance in a relationship; the obverse of dependence
Dependence	The extent that an organization relies on another organization to obtain resources that it requires to exist (e.g., material, human resources; legitimacy); the obverse of power
Adaptability	Ability of an organization to change in an attempt to address environmental demands
Demand for resources acquisition	An organization's need to acquire resources from the external environment to sustain its internal environment
**Sociotechnical theory**	
External subsystems	Outside forces and influences on an organization (e.g., stakeholders; regulations)
Social subsystems	Attributes of people (i.e., skills, attitudes, concerns, expectation, and values); relationships among people; reward systems; and authority structure
Technical subsystems	Technologies, techniques, tasks performance, methods and work setting; features include data cleansing and migration, features and functionalities of application, adaptability and flexibility or new system, system benefits, usability, stability
Organizational subsystems	Infrastructure, leadership and management, resources, teamwork and communication, organizational readiness for change, organizational context
Interdependence	The interaction among social subsystems, technical subsystems, and organizational subsystems
**Transaction cost economics**	
Asset specificity (of transactions)	The degree to which transacting parties have invested transaction-specific human, physical, or other forms of capital specific to the transaction (e.g., additional training, equipment, and staff)
Uncertainty	The extent to which changes to the wider environment may influence transactions and the future actions of transacting parties are unknown
Frequency (of transactions)	How often a transaction occurs
Transaction costs	The outlay required for contract negotiations, monitoring adherence to contractual terms, providing financial incentives or penalties, and losses resulting from supplier noncompliance
Governance structure	∙ A continuum of approaches to generating a desired product or service ranging from buying it from another party to making it yourself: Spot market is when organization buys with no contract (i.e., open market) ∙ ”Hybrid" contracting modes are when organization buys with a contract, and may include long-term commercial contracts, informal agreements, and franchise contracting, exclusive dealing contract ∙ Fully integrated firm is when the organization makes the product itself, by unifying ownership and control

#### Theory Overview

The theory overview section provides a brief orientation to the theory's basic tenets. For example, the overview of transaction cost economics theory is: ‘Organizations incur costs as a result of planning, implementing, and enforcing transactions with other organizations. Organizations strive for greater efficiency by implementing governance structures that will minimize transaction costs.'

#### Example Application to Implementation Science

In this section, we provide full citations of empirical studies that used the organization theory. For example, for complexity theory, we provide references for two studies: Braithwaite et al.'s (32) 2018 empirical analysis of systems change and Colón-Emeric et al.'s (33) 2017 cluster-randomized trial of an intervention to improve staff interactions around fall prevention in nursing homes.

#### Constructs and Definitions

In the constructs column of this section, we list each construct from the theory. In the definitions column, we provide a definition of each construct from one or more of the foundational texts, often with practical examples. For example, organizational learning theory includes the construct ‘explicit knowledge,’ which is defined as ‘facts and information that can be codified (e.g., in policies and procedures)' (34).

#### Propositions

In this section, each proposition includes at least two constructs and posits relationships among those constructs. In the case of each theory, multiple propositions are listed. Examples of propositions from resource dependency theory include: ‘competition increases uncertainty and decreases stakeholders’ willingness to adopt or implement new strategies' and ‘decreased munificence requires organizations to reduce their dependence on some resources and/or find alternative resources.’

#### Relevance to Implementation Science

Each statement of relevance to implementation science includes an implementation outcome and a construct from the theory. For example, sociotechnical systems theory proposes that implementation may be facilitated by optimizing organizational, social, and technical subsystems. Network perspective proposes that direct and indirect ties, network density, cohesion, embeddedness, and flexibility among organizations influence the diffusion, dissemination, adoption, scale-up, and spread of evidence-based practices (35).

#### Criticisms and/or Bounds on the Theory

This section lists full references for any articles that critique the theory or suggest contexts or conditions under which the theory may or may not apply, as well as examples of criticisms or bounds. For example, in her 1981 paper, Claudia Bird Schoonhoven (36) argues that contingency theory's statements are ambiguous and that the relationships it hypothesizes are of ambiguous functional form.

## Discussion

This study sought to summarize organization theories and their relevance to implementation science. Using expert-developed and –refined abstraction forms, we synthesized nine organization theories that collectively include 70 constructs and 65 propositions with relevance to implementation science. Our objective in developing the forms was to increase implementation scientists' access to organization theories. To that end, we have posted OTIS abstraction forms on the CPCRN website (22). In achieving our objective, we have made publicly available a subset of the many published organization theories that may be relevant to implementation science, introducing implementation scientists to a more comprehensive set of constructs relating to organizations' internal and external settings and nuanced explanations of organization-level influences on implementation. Increasing access to organization theories gives implementation scientists the opportunity to incorporate perspective on the power that organizations exert to adopt, implement, and sustain innovations—or to resist adopting, implementing, or sustaining innovations. The nuanced perspective that organization theories offer to implementation science is documented in several extant studies [e.g., (27, 37)]. For example, Leeman & Mark (38) applied Transaction Cost Analysis to understand health plans' decisions to exert their power to improve chronic illness management by employing a centralized team of case managers vs. incentivizing clinics to redesign the way they provide care. The benefits of applying organization theory to implementation science has also been documented (27). The increased access to organization theories that the forms offers has the potential to extend the perspective that organization theories offer more broadly in implementation science.

The organization-level constructs that we identified in our study can be used to expand the relatively limited scope of constructs represented in, for example, the CFIR's inner and outer setting domains and the TDF's environmental context and resources domain. Collectively, the CFIR's inner and outer setting domains include nine constructs, and the TDF's environmental context and resources domain includes four constructs (39). Thus, OTIS substantially broadens implementation science's conceptualization of organization-level constructs by describing 70 constructs that go beyond the organization-level constructs included in extant implementation TMFs.

OTIS abstraction forms include constructs not represented in extant implementation TMFs such as uncertainty, power, complexity, and competition. OTIS abstraction forms can be used to complement the CFIR's organization-level constructs, which are based in part on other theories, models and frameworks, and in part on evidence. The 65 OTIS propositions describe, explain, and predict relationships between organization-level constructs and implementation. OTIS abstraction forms may also enhance understanding of the relationship between the TDF's organization-level constructs, which are based in psychological theories intended to conceptualize individual behavior, with organization theories, which are specifically designed to conceptualize organization-level phenomena that influence implementation. Future work that directly maps OTIS constructs and propositions onto organization-level constructs in extant implementation TMFs is needed to broaden extant TMFs' conceptualization of organization-level constructs and to explain how organization-level constructs influence implementation.

In addition to integration with extant implementation TMFs, we hope that implementation scientists find that the abstraction forms themselves enhance their work. Implementation scientists may use the forms to develop basic understanding of organization theories relevant to implementation science; define included constructs; identify propositions and potential relevance to implementation science; and understand criticisms and/or bounds on the theories. We hope that information regarding organization theories will promote high-quality theory application in implementation science. That is, exposure to OTIS abstraction forms may help implementation scientists to evaluate whether a given organization theory is relevant to their work and, if so, to apply the theory knowledgeably and appropriately. This is an important contribution given evidence that implementation scientists have been found to superficially and inappropriately apply theories, models, and frameworks (40); appropriate theory application is essential for the selection of strategies, as theory identifies the mechanisms by which strategies produce outcomes (41). Indeed, our work has already enhanced implementation science. For example, one OTIS workgroup member applied resource dependency theory to conceptualize aspects of organizations and resources that may have been impacted by the COVID-19 pandemic (42).

There are several limitations to this study. First, our Delphi approach was limited to our network of colleagues with scholarship at the intersection of organization theory and implementation science. We may have failed to recruit participants who could have suggested theories, texts, or perspectives not reflected in our resulting abstraction forms. Second, the respondents represented a geographically-limited group of implementation scientists, only covering the U.S., Canada, and Western Europe. Third, despite our attempts to methodically abstract relevant constructs and propositions from foundational texts, we may have omitted information from the abstraction forms. Third, we may have failed to include relevant organization theories. To address this, we regard the abstraction forms that we have compiled as living documents to be revised and augmented through implementation scientists' careful review, critique, and application.

To further promote access to organization theories for implementation science, the forms have been added to the CPCRN website (22). We will also incorporate the forms into the CPCRN Scholars program with the goal of increasing knowledge and access to organization theories among an interdisciplinary audience of implementation scientists. The forms may be useful for other implementation science training programs and we hope by sharing them in these spaces, we can increase their use by implementation scientists. We are in the process of consolidating the organization theory constructs into domains and translating the resulting framework for use among policymakers and practitioners. The resulting OTIS framework may help implementation scientists to consider a more comprehensive set of factors in organizations' internal and external environments as well as determining which organization theories are relevant to their work. Finally, the forms that we developed may be useful for adding organization theories that are identified in the future, or for efforts to summarize TMFs at other levels (e.g., teams; communities).

## Conclusions

Organization theories offer implementation researchers numerous existing, highly relevant, yet largely untapped explanations of the organizational dynamics underlying the implementation of evidence-based practices. To advance the use of organization theory among implementation scientists, we summarized organization theories most relevant to implementation science. To promote the theories' relevance to the field, we invite implementation scientists to review, critique, and apply the forms, and to contribute their resulting feedback to the OTIS workgroup for incorporation into these living documents.

## Data Availability Statement

The original contributions presented in the study are included in the article/Supplementary Materials, further inquiries can be directed to the corresponding author/s.

## Author Contributions

The authors confirm contribution to the paper as follows: Study conception and design: SB, LK, and JL. Data collection and analysis and interpretation of results: SB, LK, MW, MB, PN, MC-B, and JL. Draft manuscript preparation: SB, LK, MW, CW, MB, PN, MC-B, AP, and JL. All authors reviewed the results and approved the final version of the manuscript.

## Funding

This publication was supported by the Centers for Disease Control and Prevention of the U.S. Department of Health and Human Services (HHS) as part of a financial assistance award with 100 percent funded by CDC/HHS (Cooperative Agreement Number U48 DP006400). The contents are those of the author(s) and do not necessarily represent the official views of, nor an endorsement, by CDC/HHS, or the U.S. Government.

## Author Disclaimer

The contents are those of the author(s) and do not necessarily represent the official views of, nor an endorsement, by CDC/HHS, or the U.S. Government.

## Conflict of Interest

The authors declare that the research was conducted in the absence of any commercial or financial relationships that could be construed as a potential conflict of interest.

## Publisher's Note

All claims expressed in this article are solely those of the authors and do not necessarily represent those of their affiliated organizations, or those of the publisher, the editors and the reviewers. Any product that may be evaluated in this article, or claim that may be made by its manufacturer, is not guaranteed or endorsed by the publisher.

## References

[1] Denis JL Hébert Y Langley A Lozeau D Trottier L-H. Explaining diffusion patterns for complex health care innovations. Health Care Manage Rev. (2002) 27:60–73. 10.1097/00004010-200207000-00007 12146784

[2] Fraser I. Translation research: where do we go from here? Worldview Evid-Bas Nurs. (2004) 1:S78–83. 10.1111/j.1524-475X.2004.04046.x 17129340

[3] Damschroder LJ Aron DC Keith RE Kirsh SR Alexander JA Lowery JC. Fostering implementation of health services research findings into practice: a consolidated framework for advancing implementation science. Implement Sci. (2009) 4:1–15. 10.1186/1748-5908-4-50 19664226 PMC2736161

[4] Stetler CB Damschroder LJ Helfrich CD Hagedorn HJ, A. guide for applying a revised version of the PARIHS framework for implementation. Implement Sci. (2011) 6:1–10. 10.1186/1748-5908-6-99 21878092 PMC3184083

[5] Helfrich CD Weiner BJ McKinney MM Minasian L. Determinants of implementation effectiveness: adapting a framework for complex innovations. Med Care Res Rev. (2007) 64:279–303. 10.1177/1077558707299887 17507459

[6] McAdam R, A. multi-level theory of innovation implementation: normative evaluation, legitimisation and conflict. Eu J Innov Manag. (2005). 10.1108/14601060510610216

[7] Harvey G Kitson A. Promoting action on research implementation in health services: the integrated-PARIHS framework. Handbook Implement Sci: Edward Elgar Publishing. (2020) 3:12. 10.4337/9781788975995.0001233089474

[8] Klein KJ Sorra JS. The challenge of innovation implementation. Aca Manag Rev. (1996) 21:1055–80. 10.5465/amr.1996.9704071863

[9] Powell BJ Beidas RS Rubin RM Stewart RE Wolk CB Matlin SL. Applying the policy ecology framework to Philadelphia's behavioral health transformation efforts. Administ Pol Mental Health Mental Health Serv Res. (2016) 43:909–26. 10.1007/s10488-016-0733-6 27032411 PMC5045753

[10] Raghavan R Bright CL Shadoin AL. Toward a policy ecology of implementation of evidence-based practices in public mental health settings. Implement Sci. (2008) 3:1–9. 10.1186/1748-5908-3-26 18485219 PMC2396668

[11] Raghavan R Inkelas M Franke T Halfon N. Administrative barriers to the adoption of high-quality mental health services for children in foster care: a national study. Administ Pol Mental Health Mental Health Serv Res. (2007) 34:191–201. 10.1007/s10488-006-0095-6 17211714

[12] Daft RL. Organization Theory and Design. Cincinnati, Ohio: South-Western College Publishing (2004).

[13] Daft RL. Management. San Diego, CA: Harcourt College Publishers (1997).

[14] Birken SA Bunger AC Powell BJ Turner K Clary AS Klaman SL. Organizational theory for dissemination and implementation research. Implement Sci. (2017) 12:1–15. 10.1186/s13012-017-0592-x 28499408 PMC5427584

[15] Bonner M Koch T Langmeyer D. Organizational theory applied to school reform: a critical analysis. Sch Psychol Int. (2004) 25:455–71. 10.1177/0143034304048779

[16] Forrester JP Adams GB. Budgetary reform through organizational learning: Toward an organizational theory of budgeting. Adm Soc. (1997) 28:466–88. 10.1177/009539979702800403

[17] Hunter DE. Using a theory of change approach to build organizational strength, capacity and sustainability with not-for-profit organizations in the human services sector. Eval Program Plann. (2006) 29:193–200. 10.1016/j.evalprogplan.2005.10.003

[18] Payne J Leiter J. Structuring agency: examining healthcare management in the USA and Australia using organizational theory. J Health Organ Manag. (2013). 10.1108/14777261311311825 23734479

[19] Sarkies M Robinson S Ludwick T Braithwaite J Nilsen P Aarons G. Understanding implementation science from the standpoint of health organisation and management: an interdisciplinary exploration of selected theories, models and frameworks. J Health Organ Manag. (2021). 10.1108/JHOM-02-2021-0056

[20] Jacobs SR Weiner BJ Bunger AC. Context matters: measuring implementation climate among individuals and groups. Implement Sci. (2014) 9:1–14. 10.1186/1748-5908-9-46 24742308 PMC4012549

[21] Nilsen P Potthoff S Birken SA. Conceptualising four categories of behaviours: implications for implementation strategies to achieve behaviour change. Front Health Serv. (2022) 1:795144. 10.3389/frhs.2021.795144 PMC10012728 36926485

[22] CPCRN. OTIS Workgroup abstraction forms. 2022. Available online at: https://cpcrn.org/resources?open_section=otis

[23] Vax S Farkas M Russinova Z Mueser KT Drainoni M-L. Enhancing organizational readiness for implementation: constructing a typology of readiness-development strategies using a modified Delphi process. Implement Sci. (2021) 16:1–11. 10.1186/s13012-021-01132-0 34112191 PMC8194182

[24] Elwyn G O'Connor A Stacey D Volk R Edwards A Coulter A. Developing a quality criteria framework for patient decision aids: online international Delphi consensus process. BMJ. (2006) 333:417. 10.1136/bmj.38926.629329.AE 16908462 PMC1553508

[25] Okoli C Pawlowski SD. The Delphi method as a research tool: an example, design considerations and applications. Inform Manag. (2004) 42:15–29. 10.1016/j.im.2003.11.002

[26] Health A. 12th Annual Conference on the Science of Dissemination and Implementation in Health 2019. Available online at: https://academyhealth.org/events/2019-12/12th-annual-conference-science-dissemination-and-implementation-health

[27] Leeman J Baquero B Bender M Choy-Brown M Ko LK Nilsen P. Advancing the use of organization theory in implementation science. Prevent Med. (2019) 129:105832. 10.1016/j.ypmed.2019.105832 31521385 PMC7076554

[28] Plsek PE Wilson T. (2001). Complexity science: Complexity, leadership, and management in healthcare organisations. The British Medical Journal, 323:746–749. 11576986 10.1136/bmj.323.7315.746 PMC1121291

[29] Powell WW DiMaggio PJ (eds.) (1991). The New Institutionalism in Organizational Analysis. Chicago, IL: Chicago University Press.

[30] Hannan MT Freeman JH. The population ecology of organizations. Am. J. Sociol. (1977) 83:929–84.

[31] Yeager DS Fong CJ Lee HY Espelage DL. Declines in efficacy of anti-bullying programs among older adolescents: Theory and a three-level meta-analysis. J Appl Dev Psychol. (2021) 75:101296. 10.1016/j.appdev.2021.101296 34737486 PMC8562654

[32] Braithwaite J Churruca K Long JC Ellis LA Herkes J. When complexity science meets implementation science: a theoretical and empirical analysis of systems change. BMC Med. (2018) 16:1–14. 10.1186/s12916-018-1057-z 29706132 PMC5925847

[33] Colón-Emeric CS Corazzini K McConnell ES Pan W Toles M Hall R. Effect of promoting high-quality staff interactions on fall prevention in nursing homes: a cluster-randomized trial. JAMA Intern Med. (2017) 177:1634–41. 10.1001/jamainternmed.2017.5073 28973516 PMC5710274

[34] Stover M. Making tacit knowledge explicit: The ready reference database as codified knowledge. Ref Serv Rev. (2004). 10.1108/00907320410537685

[35] Gulati R Gargiulo M. Where do interorganizational networks come from? Am J Sociol. (1999) 104:1439–93. 10.1086/21017929594896

[36] Schoonhoven CB. Problems with contingency theory: testing assumptions hidden within the language of contingency“ theory”. Administ Sci Quart. (1981) 5:349–77. 10.2307/2392512 10252610

[37] Lengnick-Hall R Willging C Hurlburt M Fenwick K Aarons GA. Contracting as a bridging factor linking outer and inner contexts during EBP implementation and sustainment: a prospective study across multiple US public sector service systems. Implementation Science. (2020) 15:1–16. 10.1186/s13012-020-00999-9 32527274 PMC7288508

[38] Leeman J Mark B. The chronic care model versus disease management programs: A transaction cost analysis approach. Health Care Manage Rev. (2006) 31:18–25. 10.1097/00004010-200601000-00004 16493269

[39] Birken SA Powell BJ Presseau J Kirk MA Lorencatto F Gould NJ. Combined use of the consolidated framework for implementation research (CFIR) and the theoretical domains framework (TDF): a systematic review. Implement Sci. (2017) 12:1–14. 10.1186/s13012-016-0534-z 28057049 PMC5217749

[40] Birken SA Powell BJ Shea CM Haines ER Alexis Kirk M Leeman J. Criteria for selecting implementation science theories and frameworks: results from an international survey. Implement Sci. (2017) 12:1–9. 10.1186/s13012-017-0656-y 29084566 PMC5663064

[41] Sales AE Barnaby DP Rentes VC. Letter to the editor on “the implementation research logic model: a method for planning, executing, reporting, and synthesizing implementation projects” (Smith JD, Li DH, Rafferty MR. the implementation research logic model: a method for planning, executing, reporting, and synthesizing implementation projects. Implement Sci. (2020) 15:84. 10.1186/s13012-021-01169-1 32988389 PMC7523057

[42] Hara-Hubbard K Lee EJ Lim JS Bishop S Gaffney A Wong D. Resilience of community-based organizations serving older Asian American, Native Hawaiian, and Pacific Islanders adults during the COVID-19 pandemic. Paper presented at: The American Public Health Association Annual Meeting. (2021) Oct 24–27; Virtual.

