# Supplementary material for: Increasing Access to Organization Theories for Implementation Science
Source: Front Health Serv. 2022 Jun 30;2:891507. doi: 10.3389/frhs.2022.891507 (PMC10012830; doi:10.3389/frhs.2022.891507)
Supplement: Supplementary file 1 [file Data_Sheet_1.PDF]

Overview	
Complexity science focuses on understanding how change occurs in complex adaptive systems (i.e., systems that are made up of many interdependent, heterogeneous parts that interact in a nonlinear fashion). The system may be conceptualized as a unit within an organization, the organization, and/or the wider inter-organizational system of which the organization is a part.	
**Example Application to Implementation Science**	
Braithwaite, J., Churruca, K., Long, J. C., Ellis, L. A., & Herkes, J. (2018). When complexity science meets implementation science: a theoretical and empirical analysis of systems change. *BMC medicine, 16*(1), 63.<br><br>Colón-Emeric, C. S., Corazzini, K., McConnell, E. S., Pan, W., Toles, M., Hall, R., . . . Anderson, A. L. (2017). Effect of promoting high-quality staff interactions on fall prevention in nursing homes: a cluster-randomized trial. *JAMA internal medicine, 177*(11), 1634-1641.	
**Construct**	**Definition**
Self-organization	A process whereby local interactions give rise to patterns of organization
Uncertainty	The unpredictability of a system's behavior and its effects
Interdependence	The relationships, connections, and interactions among the parts of a complex system
Feedback loops	A phenomenon characterized by outputs of a system continuously becoming the inputs
Minimum specifications	A few, flexible, simple rules:<br>1. direction pointing (accounting for past phenomena in future iterations)<br>2. boundaries (delimitations of the system)<br>3. resources (means available)<br>4. permissions (latitude in decision-making; Pslek and Wilson, 2001)
Sense making	A social activity through which people assign meaning to experience

Propositions

1. Interdependencies contribute to sense making.
2. Interdependencies among people with diverse perspectives contribute to more effective sense making
3. "Interdependencies that are trusting, attentive to new ideas, and mindful of differences between ideas are more likely to result in effective sense making than interdependencies that lack these qualities." (Lanham et al., 2009 as cited in Lanham et al., 2013)
4. Interdependencies and sense making contribute to self-organization.
5. Feedback loops may amplify some effects and reduce others.
6. At times, small changes will lead to large scale differences in outcomes (i.e., "the butterfly effect") and vice versa.
7. Change that is guided by minimum specifications allows individuals to self-organize most effectively.
8. The whole system is greater than the sum of its parts.

Potential Relevance to Implementation Science

Implementation involves as sequence of events that occur within the normal, ongoing dynamics of the organization.

Complexity theory suggests the following **implementation strategies as a means of leveraging those ongoing dynamics**– all from Lanham et al (2013):

1. Leverage existing and foster new interdependencies.

a. Assess patterns of interaction (interdependencies).
b. Attend to existing and developing interdependencies in scale-up and spread (SUS) settings.
c. Acknowledge interdependencies as critical to SUS success.
d. Assess the quality and strength of interdependencies.
e. Reinforce existing relationships when effective.
f. Foster new relationships where needed.
g. Foster trust among those who are interacting.
h. Encourage interdependent experiences to foster collective sense making.
i. Conduct cyclical small studies to foster local patterns of self-organization.
j. Fortify existing interdependencies with increased communication and novel communication channels.

2. Acknowledge lack of predictability.
    a. Allow design to be tailored to local contexts.
    b. Emphasize discovery in each intervention setting.
    c. Design for multiple plausible futures.
    d. Encourage SUS stakeholders to conceptualize surprises as opportunities.
    e. Encourage SUS participants to collectively learn and adapt during implementation (Lanham et al, 2013).

3. Recognize self-organization.
    a. Develop "good enough" SUS designs with the expectation that the design will be modified as initial plans are implemented and experience is gained.
    b. Solicit input into intervention design.
    c. Encourage sensemaking.
    d. Engage individuals with diverse perspectives.
    e. Encourage focused experimentation.
    f. Encourage participants to ask questions, admit ignorance and cope with paradox.
    g. Seek out diverse points of view.
    h. Offer opportunities for reflection and conversation. (Lanham et al, 2013).

Criticisms and/or Bounds on the Theory

Cochran-Smith, M., Ell, F., Ludlow, L., Grudnoff, L., & Aitken, G. (2014). The challenge and promise of complexity theory for teacher education research. *Teachers College Record, 116*(5), 1-38.

References

Lanham, H. J., Leykum, L. K., Taylor, B. S., McCannon, C. J., Lindberg, C., & Lester, R. T. (2013). How complexity science can inform scale-up and spread in health care: understanding the role of self organization in variation across local contexts. *Social Science & Medicine*, *93*, 194-202.

Miller, W. L., Rubinstein, E. B., Howard, J., & Crabtree, B. F. (2019). Shifting implementation science theory to empower primary care practices. *The Annals of Family Medicine, 17*(3), 250-256.

Plsek, P. E., & Wilson, T. (2001). Complexity science: Complexity, leadership, and management in healthcare organisations. *The British Medical Journal,* 323(7315), 746-749.

Waldrop, M. M. (1993). Complexity: The emerging science at the edge of order and chaos. *Simon and Schuster.* New York, New York.

**Type: Theory (grand, mid-range), perspective, model, etc.**
• Grand theory

This report is supported by the Centers for Disease Control and Prevention of the U.S. Department of Health and Human Services (HHS) as part of a financial assistance award with 100 percent funded by CDC/HHS (Cooperative Agreement Number U48 DP006400). The contents are those of the author(s) and do not necessarily represent the official views of, nor an endorsement, by CDC/HHS, or the U.S. Government.

Overview	
"To be most effective, organizational structures should be appropriate to the work performed and/or to the environmental conditions facing the organization." (Schoonhoven, 1981) In other words, the optimal way of structuring work will be contingent on characteristics of both the work being performed (i.e., the task) and the environment where the work is performed (i.e., task environment).	
**Example Application to Implementation Science**	
Leeman, J., Baquero, B., Bender, M., Choy-Brown, M., Ko, L. K., Nilsen, P., . . . Birken, S. A. (2019). Advancing the use of organization theory in implementation science. *Preventive medicine, 129*, 105832.	
**Construct**	**Definition**
Task	The work that is performed
Task environment	The context where work is performed (both the organizational setting and its wider, socio-political-economic context)
Uncertainty in the task or task environment	The gap between the amount of information that is needed and the amount of information that is available to achieve a given level of performance on a task<br><br>Factors that may contribute to uncertainty include:<br>• Rate of technical change (how rapidly is the technology required to complete a task changing?)<br>• Lack of information about the availability of resources and stakeholder preferences and demand<br>• Strength/quality of evidence in support of a tasks' impact on intended outcomes
How a task/work is structured: Programmed versus un-programmed coordination (integration)	• Programmed coordination: The activities involved in completing a task are specified and codified in advance via (1) rules and programs (i.e., standardization) and (2) centralization of decision making and authority arrangements<br>• Unprogrammed coordination: The activities involved in completing a task are not specified in advance by the organization; activities are worked out by organization members via (1) professionalization deferring to expertise, (2) providing additional time and resources for collaboration, (3) creation of self-contained tasks, (4) providing real-time data to frontline individuals and teams, and (5) promoting and supporting horizontal coordination and communication
Interdependence	To what degree/extent different actors must interact to complete work.
Differentiation	The extent to which, within an organization, different parts/departments perform different tasks and have different relevant sub-environments.
**Propositions**	

1. The optimal structure of work is contingent on the uncertainty of the task and task environment: When uncertainty is higher, unprogrammed means of coordination will be the more effective way to structure a task; when uncertainty is low, programmed means of coordination will be more effective.
2. Higher levels of interdependence (both within and between departments) will require greater investment in coordination (integration).
3. The greater the differentiation between departments, the more difficult it will be to coordinate (integrate).

Potential Relevance to Implementation Science

1. Contingency theory suggests that **implementation strategies** should include assessing (1) uncertainty and interdependence related to the task and (2) uncertainty in the task environment.
2. If uncertainty is low, the **adoption decision** should favor a standardized, manualized, prescriptive evidence-based intervention.
3. If uncertainty is low, **implementation strategies** should seek to standardize implementation (e.g., mandate change).
4. If uncertainty is high, the **adoption decision** should favor more flexible EBIs and/or menus of EBIs
5. If uncertainty is high, **implementation strategies** should seek to promote coordination and communication (e.g., audit and provide feedback; build a coalition; create new clinical teams).
6. If the task requires interdependent interactions between departments, **implementation strategies** should strengthen coordination and communication between departments (e.g., implementation teams, local consensus discussion, cyclical small tests of change).

Criticisms and/or Bounds on the Theory

Schoonhoven, C. B. (1981). Problems with contingency theory: testing assumptions hidden within the language of contingency" theory". *Administrative science quarterly*, 349-377.

1. “Ambiguous character of the ‘theoretical’ statements
2. Implied hypothesized interactions among variables
3. Unspecified functional form of hypothesized interactions
4. Potentially misplaced assumption of linearity of hypothesized relationships
5. Potentially misplaced assumption of symmetrical relationships among constructs (a change in independent variable produces and equal and symmetrical change in the dependent variable)

Donaldson L. The Contingency Theory of Organizations. Foundations for Organizational Science. 2001

References

Burns, T., & Stalker, G. M. (1961). The management of innovation. London. *Tavistock Publishing. Cited in Hurley, RF and Hult, GTM (1998). Innovation, Market Orientation, and Organisational Learning: An Integration and Empirical Examination. Journal of Marketing, 62*, 42-54.

Golembiewski, R. T. (1983). Professionalization, performance, and protectionism: A contingency view. *Public Productivity Review*, 251-268.

Kim, K. K. (1988). Organizational coordination and performance in hospital accounting information systems: An empirical investigation. *Accounting Review*, 472-489.

Lawrence, P. R., & Lorsch, J. W. (1967). Differentiation and integration in complex organizations. *Administrative science quarterly*, 1-47.

Nohria, N., & Gulati, R. (1996). Is slack good or bad for innovation? *Academy of management Journal, 39*(5), 1245-1264.

Type: Theory (grand, mid-range), perspective, model, etc.

- Mid-range theory

PREVENTION RESEARCH CENTERS
This report is supported by the Centers for Disease Control and Prevention of the U.S. Department of Health and Human Services (HHS) as part of a financial assistance award with 100 percent funded by CDC/HHS (Cooperative Agreement Number U48 DP006400). The contents are those of the author(s) and do not necessarily represent the official views of, nor an endorsement, by CDC/HHS, or the U.S. Government.

Overview

Institutional theory answers the question: Why do organizations tend to look so similar (i.e., exhibit isomorphism)? The degree of isomorphism in an organizational field is positively related to the degree of (1) coercive, (2) mimetic, and (3) normative pressures in the field.

Application to Implementation Science

Jensen, T. B., Kjærgaard, A., & Svejvig, P. (2009). Using institutional theory with sensemaking theory: a case study of information system implementation in healthcare. *Journal of Information Technology, 24*(4), 343-353.

Nilsen, P., Ståhl, C., Roback, K., & Cairney, P. (2013). Never the twain shall meet? -a comparison of implementation science and policy implementation research. *Implementation Science, 8*(1), 63.

Novotná, G., Dobbins, M., & Henderson, J. (2012). Institutionalization of evidence-informed practices in healthcare settings. *Implementation Science, 7*(1), 112.

Constructs

Isomorphism	Similar organizational structures and processes (dependent variable)
Coercive pressures	“Formal and informal pressures exerted on organizations by other organizations upon which they are dependent and by cultural expectations in the society within which organizations function" (DiMaggio & Powell,1983, p.150)
Mimetic pressures	Influences encouraging organizations to model the behavior of other organizations in their field
Normative pressures	Influences derived from members of an occupation or profession (e.g., physicians) defining the conditions and methods of work
Professionalization	Claims on knowledge among professional groups

Propositions

1. The degree of isomorphism in an organizational field is positively related to the degree of (1) coercive, (2) mimetic, and (3) normative pressures in that field.
2. Coercive pressures are greater to the extent that:
   - Organizations in a field transact with agencies of the state (or depend on public financing).
   - Organizations in a field are dependent upon a single (or several similar) source of support for vital resources.
3. Mimetic pressures are greater when an organizational field has high levels of uncertainty (e.g., evidence for what is effective is limited, technologies are poorly understood, goals are ambiguous, etc.).
4. Normative processes are greater in organizations with higher levels of professionalization.

Potential Relevance to Implementation Science

Implementation strategies should take advantage of existing or potential coercive, mimetic, and normative pressures on the focal organization.

1. Coercive pressures may be augmented by specifying how an intervention can assist an organization in meeting regulatory, reimbursing, or accrediting body requirements.
2. Coercive pressures may be augmented by negotiating with centralized sources of vital support (payers, suppliers).
3. Coercive pressures may be augmented by changing the policy governing reimbursement, formularies, accreditation, etc.
4. Coercive and mimetic pressures may be leveraged by creating a system to publicly recognize organizations that fully implement an intervention

5. Mimetic pressures may be augmented by partnering with opinion leading organizations to be early **adopters** and serve as models for other organizations in the field.
6. Mimetic pressures may be augmented by aligning with existing ways of improving practice that have already **diffused** (e.g., the Improvement Model, Lean, Six Sigma, etc.).
7. Normative pressures may be augmented by partnering with professional associations to support implementation.
8. Normative pressures may be augmented by strategies that increase the alignment (real or perceived) between EBI usage and professional identity/role.

Criticisms and/or bounds on the theory

Dolan, P., & Connolly, J. (2018). Beyond logic and norms: a figurational critique of institutional theory in organisation studies. *Cambio, 7*(14), 139-149.

Suddaby, R. (2010). Challenges for institutional theory. *Journal of management inquiry, 19*(1), 14-20.

References

DiMaggio, P. J., & Powell, W. W. (1983). The Iron Cage Revisited: Institutional Isomorphism and Collective Rationality in Organizational Fields. *American Sociological Review*, *48*(2), 147–160. https://doi.org/10.2307/2095101

Heugens, P. P. M. A. R., & Lander, M. W. (2009). Structure! agency! (and other quarrels): A meta-analysis of institutional theories of organization. *Academy of Management Journal*, *52*(1), 61–85. https://doi.org/10.5465/AMJ.2009.36461835

Meyer, J. W., & Rowan, B. (1977). Institutionalized Organizations: Formal Structure as Myth and Ceremony. *American Journal of Sociology*, *83*(2), 340–363. https://doi.org/10.2307/2778293\

Scott, W. R. (2005). *Encyclopedia of Social Theory* (G. Ritzer, ed.). Thousand Oaks: SAGE Publications, Inc.

Zucker, L. G. (1987). Institutional Theories of Organization. *Annual Review of Sociology*, *13*, 443–464. Retrieved from https://www.jstor.org/stable/2083256

Type: Theory (grand, mid-range), perspective, model, etc.

- Mid-range theory

PRC PREVENTION RESEARCH CENTERS
This report is supported by the Centers for Disease Control and Prevention of the U.S. Department of Health and Human Services (HHS) as part of a financial assistance award with 100 percent funded by CDC/HHS (Cooperative Agreement Number U48 DP006400). The contents are those of the author(s) and do not necessarily represent the official views of, nor an endorsement, by CDC/HHS, or the U.S. Government.

Overview

Network perspectives elucidate the social relations between actors (e.g., organizations; individuals within organizations) and how the nature and structure of those relations contribute to the actors' performance and behavior. Network perspectives explain how and why information and resources flow, and are shared, amongst a population of actors through their connections.

Example Application to Implementation Science

Burmaoglu, S., Saritas, O., Kıdak, L. B., & Berber, İ. C. (2017). Evolution of connected health: a network perspective. *Scientometrics, 112*(3), 1419-1438.

Mikhailova, O. (2018). Adoption and implementation of new technologies in hospitals: a network perspective. *IMP Journal*.

Construct	Definition
Social network	A set of actors (e.g., individuals, organizations) connected by one or more social ties (e.g., advice ties, friendship ties)
Direct ties	Connections in which a single tie spans two actors
Indirect ties	Connections where ties exist between actors but only through other actors
Patterns of relations	Patterns of ties that yield a particular network structure (e.g., structural holes)
Strength	Amount of time, emotional intensity, intimacy (mutual confiding) and reciprocity of the tie
Centrality	The importance of an actor's position in a network structure (e.g., prominence of opinion leaders)
Cohesion	The connectedness or "knitted-ness" of a network
Network density	A measure of cohesion expressed as the number of ties in a network divided by the maximum number of ties that are possible
Constraint	A linkage or other restriction that becomes a limitation and/or an inhibition
Embeddedness	The extent that social ties are forged, renewed, and extended through the community rather than through actors outside the community
Flexibility	The extent that social ties are forged, renewed, and extended through the community rather than through actors outside the community

Propositions

1. The more organizations, holding ties constant, the lower the network density.
2. Direct and indirect ties (but particularly the latter) increase flexibility in access to what is flowing through network ties (e.g., information).
3. Central actors, on average, receive what is flowing through network ties (e.g., information) sooner than other actors.
4. Influence flows across direct and indirect ties among organizations within a network.
5. Fewer indirect ties will limit connections with other organization(s).

Relevance to Implementation Science

1. Direct and indirect ties, network density, cohesion, embeddedness, and flexibility *among organizations* affects **diffusion**, **dissemination**, **adoption**, **scale-up**, and **spread** of EBPs.
2. Direct and indirect ties, network density, cohesion, embeddedness, and flexibility among individuals *within organizations* affects **implementation** and **sustainment** of EBPs.

3. Network operation skill moderates the influence of direct and indirect ties, network density, cohesion, embeddedness, and flexibility on the aforementioned **implementation outcomes**—i.e., improved network operation skill augments ties' spread of EBPs.
4. Ties can be created (to bridge structural holes) and strengthened with **implementation strategies** such as bridging factors, building coalitions, building local consensus building, embedding opinion leaders, and developing advisory boards and workgroups.

Parameters

Criticisms and/or Bounds on the Theory

None identified

References

Borgatti, S. P., & Halgin, D. S. (2011). On network theory. *Organization Science*, *22*(5), 1168–1181. https://doi.org/10.1287/orsc.1100.0641

Granovetter, M. S. (1973). The strength of weak ties. *American journal of sociology, 78*(6), 1360-1380.

Kilduff, M., & Brass, D. J. (2010). Organizational Social Network Research: *Core Ideas and Key Debates*. *The Academy of Management Annals*, *4*(1), 317–357. https://doi.org/10.1080/19416520.2010.494827

Podolny, J. M., & Page, K. L. (1998). Network Forms of Organization. *Annual Review of Sociology*, *24*(1), 57–76. https://doi.org/10.1146/annurev.soc.24.1.57

PRC PREVENTION RESEARCH CENTERS

This report is supported by the Centers for Disease Control and Prevention of the U.S. Department of Health and Human Services (HHS) as part of a financial assistance award with 100 percent funded by CDC/HHS (Cooperative Agreement Number U48 DP006400). The contents are those of the author(s) and do not necessarily represent the official views of, nor an endorsement, by CDC/HHS, or the U.S. Government.

Overview
Organizational learning is the process of creating, retaining, and transferring knowledge within an organization. An organization improves over time as it gains experience.

Example Application to Implementation Science
Berta, W., Cranley, L., Dearing, J. W., Dogherty, E. J., Squires, J. E., & Estabrooks, C. A. (2015). Why (we think) facilitation works: insights from organizational learning theory. *Implementation Science, 10*(1), 1-13.<br><br>Tucker, A. L., Nembhard, I. M., & Edmondson, A. C. (2007). Implementing new practices: An empirical study of organizational learning in hospital intensive care units. *Management science, 53*(6), 894-907.

Construct	Definition
Explicit knowledge	Facts and information that can be codified (e.g., in policies and procedures)
Tacit knowledge	Facts, information, and skills that are difficult to codify
Learning process	An interaction of experience (history) and context that produces knowledge
Learning subprocesses	A series of actions associated with the learning process, including:<br>1. Knowledge creation: knowledge acquired from direct experience of unit (e.g., trial and error experimentation)<br>2. Knowledge transfer: knowledge transmitted through socialization, education, imitation, professionalization, personnel movement, mergers, acquisitions (Levitt & March)<br>3. Knowledge retention: knowledge that is embedded in active context (e.g., written policies; job roles)<br>4. Knowledge search: seeking solutions (in the form of information) for organizational problems
Dominance of organization in field of competitors	The extent to which an organization is perceived to be powerful in relation to its competitors
Complexity of an organization's environment	The extent to which the context in which an organization operates is or is not (1) stable over time and (2) predictable (e.g., customer preferences; availability of resources)

Propositions

1. Organizational learning is a process that occurs over time, in cycles, with varying frequency and timing (before, during, or after task performance) across multiple systems levels (individual, group, organization, and inter-organizational), and within a context that includes the organization and the environment in which the organization is embedded (Argote and Miron-Spector, 2011).
2. Organizational learning occurs through changes in cognitions or behavior.
3. Organizational learning includes both explicit and tacit components.
4. Organizational learning may be active (i.e., strategic) or passive (e.g., by accident).
5. Organizational learning requires both change (to introduce new information) and stability (to facilitate interpreting information).
6. Organizations that are resistant to the change required for learning are vulnerable to paradigm peddling (e.g., promises of positive learning outcomes) and paradigm politics (e.g., posturing for acceptance of one mode of achieving positive learning outcomes) (Levitt and March, 1988).
7. Organizational learning can occur naturally, or it can be simulated.
8. Organizational learning can occur at higher- or lower-order levels. Double loop learning occurs when the organization's underlying norms, policies and objectives are changed (higher-level). Single loop

learning occurs when the organization's fixes a problem but does not address the underlying norms, policies and objectives that contributed to the problem.

9. Knowledge derived from organizational learning may be embedded in active context (e.g., routines, tools, tasks) and in latent context (i.e., invisible but nonetheless present; e.g., organizational culture).
10. Knowledge derived from organizational learning can be characterized by level of causal ambiguity (i.e., extent to which cause-effect relationship is understood).
11. Organizational learning can occur through multiple subprocesses (attentive, controlled versus routine, automatic).
12. Organizational learning subprocesses vary in their distribution across organizational members (i.e., whether learning spreads from bottom [i.e., frontline employees]-up [i.e., to top managers] versus top-down).
13. Some organizations are powerful enough to create their own environments; weaker organizations will learn to adapt to the dominant ones (i.e., they will learn to learn) (Levitt and March, 1988).
14. Powerful organizations, by virtue of their ability to ignore competition, will be less inclined to learn from experience and less competent at doing so (Levitt and March, 1988).
15. Overly complex organizational environments inhibit learning because:
    a. Environmental complexity makes establishing causality and interpreting outcomes from learning difficult.
    b. Complexity increases uncertainty, challenging perceptions and interpretations of the environment necessary for learning.
16. Stable and predictable environments favor maintaining existing routines and limit learning.
17. Overly unpredictable environments stimulate much action/change but little learning.
18. Unstable environments require renewal and innovation that meaningful learning can produce.
19. Moderately unstable environments may be the most conducive to change and subsequent learning.

Potential Relevance to Implementation Science

1. Organizations may **adopt** EBPs that highly successful organizations are using and bypass the need for direct experience; this can lead to incomplete or flawed learning.
2. **Implementation strategies** should facilitate organizational learning (e.g., through iterative, small tests of change like Plan-Do-Study-Act cycles).
3. **Implementation strategies** may also promote knowledge transfer across organizations (e.g., peer networking, learning collaboratives).
4. **Planning for implementation** should include assessing the competitive environment and identify which organizations are more and less dominant.
5. **Planning for implementation** should involve assessing the level uncertainty in the organizational environment. High levels of either uncertainty or stability may inhibit learning.
6. **Implementation outcomes** may manifest in explicit (e.g., policies) and tacit (e.g., climate) knowledge, so both types should be assessed.
7. **Implementation** may be influenced by explicit (e.g., policies) and tacit (e.g., climate) knowledge. For example, tacit knowledge of past failures (and thus reluctance to engage in change required to learn) may inhibit implementation. Assessing these factors and accounting for them may facilitate implementation.

Criticisms and/or Bounds on the Theory

Caldwell, R. (2012). Systems thinking, organizational change and agency: A practice theory critique of Senge's learning organization. *Journal of change management, 12*(2), 145-164.

Easterby-Smith, M., Araujo, L., & Burgoyne, J. (1999). *Organizational learning and the learning organization: Developments in theory and practice*: Sage.

References
Argote, L., & Minor-Spektor, E. (2011). Organizational Learning: From Experience to Knowledge. Organization Science, 22(5), 1123-1137. http://dx.doi.org/10.1287/orsc.1100.0621<br><br>Levitt, B., & March, J.G. (1988). Organizational Learning. Annual Review of Sociology, 14(319), 40.
**Type: Theory (grand, mid-range), perspective, model, etc.**
• Grand theory

This report is supported by the Centers for Disease Control and Prevention of the U.S. Department of Health and Human Services (HHS) as part of a financial assistance award with 100 percent funded by CDC/HHS (Cooperative Agreement Number U48 DP006400). The contents are those of the author(s) and do not necessarily represent the official views of, nor an endorsement, by CDC/HHS, or the U.S. Government.

Overview
Population ecology aims to understand why there are so many kinds of organizations and how organizational populations form, become different, and remain different over time (Baum, 1997). Population ecology focuses on the demographic (e.g., age, size), ecological (e.g., niche-width theory, population density), and environmental (e.g., social, economic, political, and technological) processes posited to influence the survival of organizations in a field.
**Example Application to Implementation Science**
Hovmand, P. S., & Gillespie, D. F. (2010). Implementation of evidence-based practice and organizational performance. *The journal of behavioral health services & research, 37*(1), 79-94.<br><br>Vest, J. R., & Menachemi, N. (2019). A population ecology perspective on the functioning and future of health information organizations. *Health care management review, 44*(4), 344-355.

Construct	Definition
Competition	A process by which "(1) demand for resources exceeds supply; (2) competitors become more similar as standard conditions of competition produce a uniform response; (3) selection eliminates the weakest competitors; and (4) deposed competitors differentiate either territorially or functionally, yielding a more complex division of labor" (Hannan & Freeman, 2002)
Niche/niche width	(The size of) An area in a constraint space in which a population can survive and reproduce itself
Institutional linkages	Relationships created between organization(s) for a cause
Spatial variation	Different values of organizational characteristics across locations
Technology cycles	A sequence of processes that involve technology (i.e., the means, activities, and knowledge to transform materials and inputs into outputs; e.g., human resources)
Selection pressure	External agents that affect an organization's ability to survive in a given environment
Isomorphism	A similarity of processes or structure among organizations
Community interdependence	The extent to which interactions among co-acting sets of organizational/community populations depend on each other
Stability	The extent to which conditions change over time
Population density	The number of organizations in a population (i.e., group of organizations that is distinguishable from other groups)
Internal arrangements	Actions and factors within an organization (e.g., internal politics)
Resource acquisition	The process by which new organization(s) acquire resources
Prior failures	Previous deterioration(s) in an organization's adaptation to its small niche and the associated reduction of resources within the organization
Inertia	Organizational resistance to change
Structure	An organization's goals, authority, strategy, core technology
Specialization	The restricted niche breadth/area of a given organization
Age	The length of an organization's life history
Size	The capacity to carry interactions among resources, constraints and demand
Excess capacity (or slack resources)	Production at a lower scale of output than it has been designed for

Propositions

1. Organizations can use slack resources to promote performance reliability.
2. Competition makes organizations more similar as standard conditions of competition bring forth a uniform response.
3. Competition eliminates the weakest organizations.
4. To compete, relatively weak organizations differentiate themselves territorially or functionally.
5. Niche environments encourage organizations to adapt and become successful in the absence of factors that may make survival more difficult (e.g., large niche width).
6. Technology cycles become problematic for organizations as technology becomes obsolete and new technology becomes more expensive.
7. In response to selection pressure, organizations make decisions that affect the trajectory of their future.
8. Organizations adapt their structure to best take advantage of salient features of resource environment, resulting in isomorphism.
9. Institutional linkages within an environment depends on environmental structure.
10. Stability contributes to organizational survival in the absence of other factors that may disrupt organizational homeostasis.
11. As population density increases, competition between organizations increases; and vice versa.
12. Internal arrangements can affect the stability of an organization.
13. Resource acquisition can help make an organization more competitive, more stable, and greater survival over time.
14. Unsuccessful niche adaptations reduce resources.
15. As degree of specialization increases, organizations adapt to be more successful in their niche environments. This could lead to failures outside of the niche environment or if the environment changes.
16. As size increases, organizations have greater capacity to interact with environmental stimuli such as supply, demand, and manage resources and organizational constraints.
17. Depending on niche-width, excessive specialization (through intervention adoption) may put an organization at risk if they are perceived as not sufficiently generalist (i.e., relevant to stakeholders such as patients, providers, professional orgs, etc.).

Relevance to Implementation Science

1. Organizations are focused on survival, making intervention **adoption** and **implementation** only valuable to the extent that they help fit within the niche.
2. Structural inertia limits organizations' **adoption** and **implementation** of EBPs.
3. **Adopting** EBPs may influence organizational fit with environment; deciding to use an intervention may have implications for fit and competitiveness.
4. **Implementing** EBPs may influence organizational fit with environment; using an intervention may have implications for fit.
5. **Adopting** an intervention may help an organization to specialize and thus fit into a niche.
6. 'Linking' **implementation strategies** (e.g., community-academic partnership) can moderate risk associated with intervention **adoption and implementation.**
7. Organizational characteristics like size or age may moderate risk of EBP **adoption** and **implementation** (e.g., bigger, older organizations may incur less risk).
8. Organizations may be able to **adapt** to facilitate EBP **implementation**.
9. Organizations that can **adapt** may be more likely to **sustain** EBP use.

Criticisms and/or Bounds on the Theory

Hannan, M. T., & Freeman, J. (1977). The population ecology of organizations. *American journal of sociology, 82*(5), 929-964.

Pinto, J. A. M. (2005). The population ecology paradigm: Review and critique. *Journal of Business & Economics Research (JBER), 3*(10).

Young, R. C. (1988). Is population ecology a useful paradigm for the study of organizations? *American journal of sociology, 94*(1), 1-24.

References

Clegg, R., Hardy, C., & Nord, R. (1997). Handbook of organisation studies. *Journal of the Operational Research Society, 48*(9), 962-962.

Dixit, Sunil K; Sambasivan Murali. An Integrated Multitheoretical Perspective of Public Healthcare Services Delivery Organizations Public Organization Review; Dordrecht Vol. 20, Iss. 2, (Jun 2020): 319-335.

Salimath, Manjula S; Jones, Raymond, III Population ecology theory: implications for sustainability. Management Decision; London Vol. 49, Iss. 6, (2011): 874-910

Wilbon, A. D. (2015). Technology Strategy and organizational learning: Applying population ecology to understanding the influence on firm survival. *Academy of Strategic Management Journal, 14*(2), 221.

PREVENTION RESEARCH CENTERS

This report is supported by the Centers for Disease Control and Prevention of the U.S. Department of Health and Human Services (HHS) as part of a financial assistance award with 100 percent funded by CDC/HHS (Cooperative Agreement Number U48 DP006400). The contents are those of the author(s) and do not necessarily represent the official views of, nor an endorsement, by CDC/HHS, or the U.S. Government.

Overview	
Organizations become more powerful when they can balance the benefits of acquiring necessary resources from external organizations against the dependence that comes with having to acquire resources from external organizations.	
**Example Application to Implementation Science**	
Lengnick-Hall, R., Willging, C., Hurlburt, M., Fenwick, K., & Aarons, G. A. (2020). Contracting as a bridging factor linking outer and inner contexts during EBP implementation and sustainment: a prospective study across multiple US public sector service systems. *Implementation Science, 15*(1), 1-16.<br><br>Zinn, J. S., Weech, R. J., & Brannon, D. (1998). Resource dependence and institutional elements in nursing home TQM adoption. *Health Services Research, 33*(2 Pt 1), 261.	
**Construct**	**Definition**
Munificence	The availability and accessibility of resources necessary for an organization's development and survival within the external environment
Dynamism	The rate of environmental change or innovation in the external environment
Competition	The number and diversity of stakeholders (competitors, suppliers, and buyers) that an organization needs to consider in formulating strategies (Yeager et al., 2015); perceptions that another organization in the field poses a threat
Power	Dominance in a relationship; the obverse of dependence
Dependence	The extent that an organization relies on another organization to obtain resources that it requires to exist (e.g., material, human resources; legitimacy); the obverse of power
Adaptability	Ability of an organization to change in an attempt to address environmental demands
Demand for resources acquisition	An organization's need to acquire resources from the external environment to sustain its internal environment
**Propositions**	
1. To acquire power, organizations exchange their autonomy for resources from other organizations within their field. That is, organizations want autonomy and/or control, but they need resources to survive and/or produce in a way that satisfies stakeholder demands.<br>2. Competition increases uncertainty and decreases stakeholders' willingness to adopt or implement new strategies.<br>3. Decreased munificence requires organizations to reduce their dependence on some resources and/or find alternative resources.	
**Potential Relevance to Implementation Science**	
1. Organizations may **adopt** a new strategy to adapt to dynamism in the environment or to create a more stable, predictable existence.<br>2. Making evidence-based practices (EBPs) a resource that confers power and/or decreases dependence on other organizations will increase **adoption** and/or **implementation**. Regulations or requirements can be leveraged to control the adoption (or non-adoption) of EBPs. The following features of organizations or their environment moderate this relationship:<br>a. Decreased munificence may compel organizations to adopt or implement an EBP because a lack of other resources decreases their power.<br>b. Increased competition may compel organizations to adopt or implement an EBP due to the threat of relatively less power associated with not doing so.	

c. Decreased power may compel organizations to adopt or implement an EBP because of the threat to the organization's survival implied in diminished power. But organizations with copious amounts of power may forgo accreditation because accreditation does not represent an adequate relative increase in resources.
d. Increased interdependence may compel organizations to adopt or implement an EBP because a not doing so may decrease their power within the field.
e. Increased adaptability may improve organizations' ability to adopt or implement an EBP.

3. The features of 2 above suggest the following **strategies may facilitate implementation**:
   a. Coopting entities that have resources necessary to implement.
   b. Modifying demands by, for example, getting on boards of regulatory organizations.
   c. Coordinating with other organizations to promote interdependence.
   d. Improving adaptability (e.g., by increasing slack resources).

Criticisms and/or Bounds on the Theory

Casciaro, T., & Piskorski, M. J. (2005). Power imbalance, mutual dependence, and constraint absorption: A closer look at resource dependence theory. *Administrative science quarterly, 50*(2), 167-199.

Hillman, A. J., Withers, M. C., & Collins, B. J. (2009). Resource dependence theory: A review. *Journal of management, 35*(6), 1404-1427.

Johnson Jr, B. L. (1995). Resource Dependence Theory: A Political Economy Model of Organizations. [dissertation].

References

Pfeffer, J. (2005). *Great Minds in Management: The Process of Theory Development* (K. G. Smith & M. A. Hitt, Eds.). https://doi.org/10.5465/amr.2007.23467624

Pfeffer, J., & Salancik, G. R. (1978). *The External Control of Organizations: A Resource Dependence Perspective.*

Type: Theory (grand, mid-range), perspective, model, etc.

- Mid-range theory

PRC PREVENTION RESEARCH CENTERS

This report is supported by the Centers for Disease Control and Prevention of the U.S. Department of Health and Human Services (HHS) as part of a financial assistance award with 100 percent funded by CDC/HHS (Cooperative Agreement Number U48 DP006400). The contents are those of the author(s) and do not necessarily represent the official views of, nor an endorsement, by CDC/HHS, or the U.S. Government.

Overview	
Social, technical, and organizational subsystems are interrelated parts of one system. Dynamics and mutual influences exist among the three subsystems, giving rise to the system.	
**Example Application to Implementation Science**	
McDonald, K. M., Su, G., Lisker, S., Patterson, E. S., & Sarkar, U. (2017). Implementation science for ambulatory care safety: a novel method to develop context-sensitive interventions to reduce quality gaps in monitoring high-risk patients. *Implementation Science, 12*(1), 79.<br><br>Westbrook, J. I., Braithwaite, J., Georgiou, A., Ampt, A., Creswick, N., Coiera, E., & Iedema, R. (2007). Multimethod evaluation of information and communication technologies in health in the context of wicked problems and sociotechnical theory. *Journal of the American Medical Informatics Association, 14*(6), 746-755.	
**Construct**	**Definition**
External subsystems	Outside forces and influences on an organization (e.g., stakeholders; regulations)
Social subsystems	Attributes of people (i.e., skills, attitudes, concerns, expectation, and values); relationships among people; reward systems; and authority structure
Technical subsystems	Technologies, techniques, tasks performance, methods and work setting; features include data cleansing and migration, features and functionalities of application, adaptability and flexibility or new system, system benefits, usability, stability
Organizational subsystems	Infrastructure, leadership and management, resources, teamwork and communication, organizational readiness for change, organizational context
Interdependence	The interaction among social subsystems, technical subsystems, and organizational subsystems

Propositions

1. As subsystems are individually optimized and mutually aligned, organizational performance increases.
2. The successful adoption of new technology depends on optimizing and aligning subsystems.

Potential Relevance to Implementation Science

1. **Implementation** may be facilitated by optimizing individual subsystems:
    a. Organizational subsystems: Assess and augment…
        i. Readiness to implement the evidence-based practice (EBP) (e.g., through planning strategies)
        ii. Infrastructure to implement the EBP
        iii. Leadership and management for implementing the EBP (e.g., with champions)
        iv. Communication around the EBP (e.g., Plan-Do-Study-Act cycles; learning, evaluating, and sharing lessons learned)
    b. Social subsystems: Assess and augment…
        i. Stakeholder attributes and align stakeholder attributes with EBP (e.g., improving stakeholder skills through training and technical support for EBP use; increase expectations through requirements for EBP use)
        ii. Create opportunities for social groups to interact around the EBP (e.g., learning collaboratives)
    c. Technical subsystems: Assess and augment:
        i. Support for data cleansing and migration
        ii. Application features and functionality usability

iii. Integration with existing systems<br>iv. Data security and confidentiality<br>2. **Implementation** may be facilitated by aligning subsystems using user-centered design
**Criticisms and/or Bounds on the Theory**
None identified
**References**
**Type: Theory (grand, mid-range), perspective, model, etc.**
• Grand theory

PREVENTION RESEARCH CENTERS
This report is supported by the Centers for Disease Control and Prevention of the U.S. Department of Health and Human Services (HHS) as part of a financial assistance award with 100 percent funded by CDC/HHS (Cooperative Agreement Number U48 DP006400). The contents are those of the author(s) and do not necessarily represent the official views of, nor an endorsement, by CDC/HHS, or the U.S. Government.

**Overview**
Organizations incur costs as a result of planning, implementing, and enforcing transactions with other organizations. Organizations strive for greater efficiency by implementing governance structures that will minimize transaction costs.
**Example Application to Implementation Science**
Leeman, J., & Mark, B. (2006). The chronic care model versus disease management programs: a transaction cost analysis approach. *Health care management review, 31*(1), 18-25.<br><br>Stiles, R. A., Mick, S. S., & Wise, C. G. (2001). The logic of transaction cost economics in health care organization theory. *Health care management review, 26*(2), 85-92.<br><br>Zinn, J. S., Mor, V., Intrator, O., Feng, Z., Angelelli, J., & Davis, J. A. (2003). The impact of the prospective payment system for skilled nursing facilities on therapy service provision: a transaction cost approach. *Health Services Research, 38*(6p1), 1467-1486.

**Construct**	**Definition**
Asset specificity (of transactions)	The degree to which transacting parties have invested transaction-specific human, physical, or other forms of capital specific to the transaction (e.g., additional training, equipment, and staff)
Uncertainty	The extent to which changes to the wider environment may influence transactions and the future actions of transacting parties are unknown
Frequency (of transactions)	How often a transaction occurs
Transaction Costs	The outlay required for contract negotiations, monitoring adherence to contractual terms, providing financial incentives or penalties, and losses resulting from supplier noncompliance
Governance Structure	• A continuum of approaches to generating a desired product or service ranging from buying it from another party to making it yourself: Spot market is when organization buys with no contract (i.e., open market)<br>• "Hybrid" contracting modes are when organization buys with a contract, and may include long-term commercial contracts, informal agreements, and franchise contracting, exclusive dealing contract<br>• Fully integrated firm is when the organization makes the product itself, by unifying ownership and control

Propositions

1. Increased environmental uncertainty requiring adaptation between exchange parties increases market or externally driven transaction costs.
2. Greater transaction frequency leads to economies of scale.
3. Organizations strive for greater efficiency by implementing governance structures that will minimize transaction costs.
4. For significant levels of asset specificity, an organization is more likely to make a product or service themselves rather than buy it based on features of transactions, specifically:
   4.1 Higher degree of uncertainty of the transaction;
   2.2 Higher complexity of the transaction;
   2.3 Lower frequency of the transaction.

Relevance to Implementation Science

Implementing an intervention often involves transactions between an organization that promotes and supports the implementation of the intervention (intermediary organizations) and to organization or other entities that implements the intervention into routine practice (delivery systems). Transaction Cost Economics (TCE) may be helpful to both of these types of organizations. TCE might be used to guide intermediary organizations in deciding whether it is more efficient for them to contract with a delivery system to implement an intervention or to implement some or all of the intervention themselves. The Leeman & Mark (2006) paper (above) provides an example of how TCE might guide a health plan's decision about whether to hire their own staff to deliver diabetes management services or contract with clinics to provide the service. Similarly, TCE might guide delivery system decisions about whether to provide an intervention themselves or contract with other organizations to provide all or pieces of the intervention. Applying TCE involves the following steps.

Do an initial assessment to understand the organizations that might interact to implement an intervention.

Then assess the characteristics of transactions between organizations. What is or would be the frequency of transactions, level of uncertainty, and asset specificity?

Then assess the level of uncertainty in the wider environment that may influence future transactions. For example, is there a possibility that the level of reimbursement for an intervention may change?

Based on the above assessments, determine the most efficient way to structure the transaction. Should one organization produce the service (e.g., hire its own staff to provide diabetes case management) or should the organization contract with other organizations to produce the service (e.g., incentivize primary care clinics to provide diabetes case management).

Implementation and sustainment may be improved by decreasing uncertainty and increasing the frequency of transactions associated with an EBP.

Criticisms and/or Bounds on the Theory

Ghoshal, S., & Moran, P. (1996). Bad for practice: A critique of the transaction cost theory. *Academy of management Review, 21*(1), 13-47.

Lacity, M. C., Willcocks, L. P., & Khan, S. (2011). Beyond transaction cost economics: towards an endogenous theory of information technology outsourcing. *The Journal of Strategic Information Systems, 20*(2), 139-157.

References

Shelanski, H. A., & Klein, P. G. (1995). Empirical research in transaction cost economics: a review and assessment. *Journal of Law, Economics, & Organization*, 335-361.

Williamson, O. E. (1981). The Economics of Organization the Transaction cost Approach.pdf. *American Journal of Sociology*, 87:548–577. https://doi.org/10.2307/2778934

PREVENTION RESEARCH CENTERS

This report is supported by the Centers for Disease Control and Prevention of the U.S. Department of Health and Human Services (HHS) as part of a financial assistance award with 100 percent funded by CDC/HHS (Cooperative Agreement Number U48 DP006400). The contents are those of the author(s) and do not necessarily represent the official views of, nor an endorsement, by CDC/HHS, or the U.S. Government.
