# Supplementary material for: Increasing Access to Organization Theories for Implementation Science
Source: Front Health Serv. 2022 Jun 30;2:891507. doi: 10.3389/frhs.2022.891507 (PMC10012830; doi:10.3389/frhs.2022.891507)
Supplement: Supplementary file 2 [file Data_Sheet_2.PDF]

Organizational Theories Survey

Start of Block: Introduction

Intro About this 5-minute survey:
The purpose of this voluntary research study is to identify organizational theories that are relevant to implementation.
Risks associated with completing the survey or participating in the discussion may include self-disclosure. You may minimize this risk by not identifying yourself or your organization on the survey and refraining from disclosing information that you feel to be compromising.
Although we do not expect any direct benefit to you, the field of implementation science may benefit from clearer understanding of organizational theories.
Completing this survey implies your consent. You may elect not to participate in the survey. There is no penalty to you or your organization for not completing the survey.
Please contact Sarah Birken with any questions about the research study: 1103-E McGavran-Greenberg Hall, CB# 7411, Chapel Hill, NC 27599-7411; Phone: (919) 445-0774; Fax: (919) 843-6308; birken@unc.edu
For questions about the rights of human subjects, please contact the Institutional Review Board: Medical School Building #52; 105 Mason Farm Road, CB# 7097, Chapel Hill, NC 27599-7097; Phone: (919) 966-3113; Fax: (919) 966-7879; irb_questions@unc.edu; Study number: 14-2532:

End of Block: Introduction

Start of Block: Block 1

Implementation research seeks to generate knowledge regarding the translation of evidence-informed interventions into routine practice in clinical and community settings (e.g., healthcare, social work, education). An example of implementation research might include a study of the consistency and proficiency with which healthcare providers use evidence-based interventions. *Organizational theories* explain phenomena such as change by explaining relationships between organizations and their environment. **The purpose of this survey is to identify organizational theories that you believe to be relevant for understanding what influences implementation in various settings.** Below, we list several organizational theories and related texts that may be relevant to implementation. **Please indicate whether or not you believe the following organizational theories are relevant to implementation.**

If you believe that an organizational theory is relevant to implementation, please list any additional texts of which you are aware.

1 **Contingency Theory:**

Donaldson, L. The normal science of structural contingency theory. In S.R. Clegg and C. Hardy (eds.), *Handbook of Organization Studies*, pp. 57-76. Thousand Oaks, CA: Sage, 1997.

Donaldson, L. *The Contingency Theory of Organizations*, pp. 245-289. Thousand Oaks, CA: Sage, 2001.

	Not at all relevant to implementation (1)	Somewhat relevant to implementation (2)	Relevant to implementation (3)	Very relevant to implementation (4)	Extremely relevant to implementation (5)
Contingency Theory (1)	○	○	○	○	○

2 If you believe that **contingency theory** is relevant to implementation, please list any additional texts of which you are aware.

____

____

____

____

____

Carry Forward Displayed Answers from "Contingency Theory: Donaldson, L. The normal science of structural contingency theory. In S.R. Clegg and C. Hardy (eds.), Handbook of Organization Studies, pp. 57-76. Thousand Oaks, CA: Sage, 1997. Donaldson, L. The Contingency Theory of Organizations, pp. 245-289. Thousand Oaks, CA: Sage, 2001. "

X→

3 **Resource Dependency Theory:**

Aldrich, H. *Organizations and Environments*, pp. 292-322. Englewood Cliffs, NJ: Prentice Hall, 1979. Ulrich, D., & Barney, J.B. Perspectives in organizations: resource dependence, efficiency, and population. *Academy of Management Review* 1984; 9(3):471-

481. Pfeffer, J. Developing resources dependence theory. In K.G. Smith and M.A. Hitt (eds.), *Great Minds in Management*, pp. 436-459. New York: Oxford, 2005.

	Not at all relevant to implementation (1)	Somewhat relevant to implementation (2)	Relevant to implementation (3)	Very relevant to implementation (4)	Extremely relevant to implementation (5)
Resource Dependency Theory (1)	○	○	○	○	○

4 If you believe that **resource dependency theory** is relevant to implementation, please list any additional texts of which you are aware.

____

____

____

____

____

Carry Forward Displayed Answers from "Contingency Theory: Donaldson, L. The normal science of structural contingency theory. In S.R. Clegg and C. Hardy (eds.), Handbook of Organization Studies, pp. 57-76. Thousand Oaks, CA: Sage, 1997. Donaldson, L. The Contingency Theory of Organizations, pp. 245-289. Thousand Oaks, CA: Sage, 2001. "

X→

5 **Transaction Cost Economics:**
Williamson, O.E. The economics of organization: the transaction cost approach. *American Journal of Sociology* 1981; 87(3):548-577. Barney, J.B. & Hesterly, W. Organizational economics: Understanding the relationship between organizations and economic analysis. In S.R. Clegg and C. Hardy (eds.), *Handbook of Organization Studies*, pp. 115-147. Thousand Oaks, CA: Sage, 1997. Stiles, R.A. & Mick, S.S. Components of

the costs of controlling quality: a transaction cost economics approach. *Hospital & Health Service Administration* 1997; 42(2):205-219.

	Not at all relevant to implementation (1)	Somewhat relevant to implementation (2)	Relevant to implementation (3)	Very relevant to implementation (4)	Extremely relevant to implementation (5)
Transaction Cost Economics (1)	○	○	○	○	○

6 If you believe that **transaction cost economics** is relevant to implementation, please list any additional texts of which you are aware.

____

____

____

____

____

Carry Forward Displayed Answers from "Contingency Theory: Donaldson, L. The normal science of structural contingency theory. In S.R. Clegg and C. Hardy (eds.), Handbook of Organization Studies, pp. 57-76. Thousand Oaks, CA: Sage, 1997. Donaldson, L. The Contingency Theory of Organizations, pp. 245-289. Thousand Oaks, CA: Sage, 2001. "

X→

7 **Organizational Learning:**

Levitt, B., & March, J.G. Organizational learning. *Annual Review of Sociology* 1988; 14:319-340. Weick, K.E., & Westley, F. Organizational learning: affirming an oxymoron. In

S.R. Clegg and C. Hardy (eds.), *Handbook of Organizational Studies*, pp. 440-458. Thousand Oaks, CA: Sage, 1997

	Not at all relevant to implementation (1)	Somewhat relevant to implementation (2)	Relevant to implementation (3)	Very relevant to implementation (4)	Extremely relevant to implementation (5)
Organizational Learning (1)	○	○	○	○	○

8 If you believe that **organizational learning** is relevant to implementation, please list any additional texts of which you are aware.

____

____

____

____

____

Carry Forward Displayed Answers from "Contingency Theory: Donaldson, L. The normal science of structural contingency theory. In S.R. Clegg and C. Hardy (eds.), Handbook of Organization Studies, pp. 57-76. Thousand Oaks, CA: Sage, 1997. Donaldson, L. The Contingency Theory of Organizations, pp. 245-289. Thousand Oaks, CA: Sage, 2001. "

X→

9 **Population Ecology:**

Hannan, M.T., & Freeman, J.H. The population ecology of organizations. *American Journal of Sociology* 1977; 83: 929-984. Tushman, M.L., & Romanelli, E. Organizational evolution: a metamorphosis model of convergence and reorientation. *Research in Organizational Behavior* 1985; 7: 171-222. Baum, J.A.C. Organizational ecology. In S.R. Clegg and C. Hardy (eds.), *Handbook of Organization Studies*, pp. 77-114. Thousand Oaks,

CA: Sage, 1997. Aldrich, H.E., & Ruef, M. *Organization Evolving*, *2nd ed.*, pp. 16-33. Thousand Oaks, CA: Sage, 2006.

	Not at all relevant to implementation (1)	Somewhat relevant to implementation (2)	Relevant to implementation (3)	Very relevant to implementation (4)	Extremely relevant to implementation (5)
Population Ecology (1)	○	○	○	○	○

10 If you believe that **population ecology** is relevant to implementation, please list any additional texts of which you are aware.

____

____

____

____

____

Carry Forward Displayed Answers from "Contingency Theory: Donaldson, L. The normal science of structural contingency theory. In S.R. Clegg and C. Hardy (eds.), Handbook of Organization Studies, pp. 57-76. Thousand Oaks, CA: Sage, 1997. Donaldson, L. The Contingency Theory of Organizations, pp. 245-289. Thousand Oaks, CA: Sage, 2001. "

X→

11 **Institutional Theory:**

DiMaggio, P. & Powell, W.W. The Iron Cage revisited: institutional isomorphism and collective rationality in organizational fields. *American Sociological Review* 1983; 48(2):147-160. DiMaggio, P. & Powell, W.W., eds. *The New Institutionalism in Organizational Analysis*, pp. 1-38. Chicago, IL: University of chicago Press, 1991. Meyer, J.W. & Rowan, B. Institutionalized organizations: formal structure as myth and ceremony. *American Journal of*

Sociology 1977; 83(2):340-363.http://faculty.washington.edu/jwilker/571/571readings/MeyerRowan.pdf

	Not at all relevant to implementation (1)	Somewhat relevant to implementation (2)	Relevant to implementation (3)	Very relevant to implementation (4)	Extremely relevant to implementation (5)
Institutional Theory (1)	○	○	○	○	○

12 If you believe that **institutional theory** is relevant to implementation, please list any additional texts of which you are aware.

____

____

____

____

____

Carry Forward Displayed Answers from "Contingency Theory: Donaldson, L. The normal science of structural contingency theory. In S.R. Clegg and C. Hardy (eds.), Handbook of Organization Studies, pp. 57-76. Thousand Oaks, CA: Sage, 1997. Donaldson, L. The Contingency Theory of Organizations, pp. 245-289. Thousand Oaks, CA: Sage, 2001. "

X→

13 **Network Perspective:**
Mizruchi, M.S. & Galaskiewicz, J. Networks of interorganizational relations. *Sociological Methods & Research* 1993; 22(1):46-70. Pfeffer, J. *New Directions for Organization Theory:*

Problems and Prospects, pp. 55-62. New York: Oxford University Press, 1997. Podolny, J.M. & Page, K.L. Network forms of organization. *Annual Review of Sociology* 1998; 24:57-76.

	Not at all relevant to implementation (1)	Somewhat relevant to implementation (2)	Relevant to implementation (3)	Very relevant to implementation (4)	Extremely relevant to implementation (5)
Network Perspective (1)	○	○	○	○	○

14 If you believe that the **network perspective** is relevant to implementation, please list any additional texts of which you are aware.

____

____

____

____

____

Carry Forward Displayed Answers from "Contingency Theory: Donaldson, L. The normal science of structural contingency theory. In S.R. Clegg and C. Hardy (eds.), Handbook of Organization Studies, pp. 57-76. Thousand Oaks, CA: Sage, 1997. Donaldson, L. The Contingency Theory of Organizations, pp. 245-289. Thousand Oaks, CA: Sage, 2001. "

X→

15 **Situated Change Theory:**

Estabrooks, C.A., Thompson, D.S., Lovely, J.E., & Hofmeyer, A. A guide to knowledge translation theory. *The Journal of Continuing Education in the Health Professions* 2006; 26:25-

36. Orlikowski, W. Improvising organizational transformation over time: a situated change perspective. *Information Systems Research* 1994; 7:63-92.

	Not at all relevant to implementation (1)	Somewhat relevant to implementation (2)	Relevant to implementation (3)	Very relevant to implementation (4)	Extremely relevant to implementation (5)
Situated Change Theory (1)	○	○	○	○	○

16 If you believe that **situated change theory** is relevant to implementation, please list any additional texts of which you are aware.

____

____

____

____

____

Carry Forward Displayed Answers from "Contingency Theory: Donaldson, L. The normal science of structural contingency theory. In S.R. Clegg and C. Hardy (eds.), Handbook of Organization Studies, pp. 57-76. Thousand Oaks, CA: Sage, 1997. Donaldson, L. The Contingency Theory of Organizations, pp. 245-289. Thousand Oaks, CA: Sage, 2001. "

X→

17 **Complexity Theory:**

Pisek, P.E., & Greenhalgh, T. The challenge of xomplexity in health care. *British*

Medical Journal 2001; 323:625-628. Waldrop, M.M. *Complexity: The Emerging Science at the Edge of Order and Chaos*. London: Viking Press, 1992.

	Not at all relevant to implementation (1)	Somewhat relevant to implementation (2)	Relevant to implementation (3)	Very relevant to implementation (4)	Extremely relevant to implementation (5)
Complexity Theory (1)	o	o	o	o	o

18 If you believe that **complexity theory** is relevant to implementation, please list any additional texts of which you are aware.

____

____

____

____

____

Q23 Please list any organizational theories that you believe are relevant to implementation but were not included in this survey. If possible, please also list any texts that describe these additional organizational theories.

____

____

____

____

____

End of Block: Block 1
